# Supplementary material for: Antarctic Slope Undercurrent and onshore heat transport driven by ice shelf melting
Source: Sci Adv. 2024 Apr 17;10(16):eadl0601. doi: 10.1126/sciadv.adl0601 (PMC11650804; doi:10.1126/sciadv.adl0601)
Supplement: 20240417-1 [file sciadv.adl0601.v1.pdf]

OCEANOGRAPHY

Antarctic Slope Undercurrent and onshore heat transport driven by ice shelf melting

Yidongfang Si[1]†*, Andrew L. Stewart[1], Alessandro Silvano[2], Alberto C. Naveira Garabato[2]

Copyright © 2024 The Authors, some rights reserved; exclusive licensee American Association for the Advancement of Science. No claim to original U.S. Government Works. Distributed under a Creative Commons Attribution License 4.0 (CC BY).

Elevated ice shelf melt rates in West Antarctica have been attributed to transport of warm Circumpolar Deep Water (CDW) onto the continental shelf via bathymetric troughs. These inflows are supplied by an eastward, subsurface slope current (referred to as the Antarctic Slope Undercurrent) that opposes the westward momentum input from local winds and tides. Despite its importance to basal melt, the mechanism via which the undercurrent forms, and thus what controls the shoreward heat transport, remains unclear. In this study, the dynamics of the undercurrent are investigated using high-resolution process-oriented simulations with coupled ocean, sea ice, and ice shelf components. It is shown that the bathymetric steering of the undercurrent toward the ice shelf is driven by upwelling of meltwater within the ice shelf cavity. Increased basal melt therefore strengthens the undercurrent and enhances onshore CDW transport, which indicates a positive feedback that may accelerate future melt of ice shelves, potentially further destabilizing the West Antarctic Ice Sheet.

INTRODUCTION

The volume loss of the West Antarctic ice shelves is accelerating, with the most rapid ice shelf thinning and iceberg calving detected in the Amundsen Sea (*1*–*4*). Circumpolar Deep Water (CDW), a water mass up to 4°C above local freezing temperature (*5*), drives strong basal melt when it intrudes beneath the ice shelves, affecting the thickness of the ice shelves and their ability to buttress the flow of the ice sheet into the ocean (*6*), leading to sea level rise (*7*). Since much of the grounded ice lies below sea level, the West Antarctic Ice Sheet is particularly vulnerable to warm water intrusion and may completely collapse in the future (*8*, *9*). To accurately predict the future of the West Antarctic Ice Sheet and global sea-level rise, it is essential to understand the mechanisms of heat delivery and the feedback between ocean circulation and ice shelf melt.

Near the Antarctic margins, wind stress and tides, the primary forces of the ice-ocean system, inject westward momentum into the slope current. Easterly local wind stress drives surface ocean currents to the west (*10*, *11*). Tidal oscillations advect positive vorticity southward to the continental shelf due to vortex squeezing and negative vorticity northward to the deep ocean due to vortex stretching, generating a residual circulation directed westward along the continental slope (*12*). Yet, counterintuitively, both observations and numerical models have confirmed the existence of eastward undercurrents along the continental shelf break of West Antarctica (*10*, *13*–*15*). Previous studies have suggested that the melt rates of the Amundsen Sea ice shelves covary with the strength of the undercurrent because, when encountering submarine troughs, the undercurrent transports CDW directly toward ice shelf cavities (*13*, *14*, *16*). Despite its importance in driving basal melt, it remains unclear what drives the eastward undercurrent at the ocean subsurface. A mechanistic understanding of how different forcings such as winds, tides, and buoyancy forcing regulate undercurrent strength and heat transport is still lacking.

Previous studies have indicated that tides, winds, meltwater, and topography may influence the variability of the West Antarctic Slope Undercurrent and ice shelf melt. Tides play a role in modulating the ice shelf melt rates by increasing turbulent ice/ocean exchange (*17*, *18*). Winds over the continental shelf and slope have been suggested to control the fluctuations in undercurrent flow (*14*, *19*) and ocean heat delivery onto the West Antarctic continental shelves (*20*), with a key role played by variability in the tropical Pacific through atmospheric teleconnections (*13*, *14*, *16*, *21*). In addition, the ice shelf melt response to perturbed winds is strongly influenced by melt-induced circulation (*22*). Glacial meltwater may drive ocean circulation over the continental shelf (*5*, *23*–*25*) by acting as a source of potential vorticity (PV) (*26*). Jourdain *et al.* (*27*) found a linear relationship between volume transport into ice shelf cavities and their melt rates in an Amundsen Sea regional model and suggested that meltwater may drive CDW toward the ice shelf. However, they did not establish the existence or direction of causality between CDW inflows and meltwater export. Further, although the baroclinic structure of the slope front implies a negative velocity shear, this alone is not sufficient to produce an eastward undercurrent; the cross-slope buoyancy gradient is weaker in West Antarctica than in various other stretches of the Antarctic continental slope that do not host undercurrents. Although the formation dynamics of undercurrents in other parts of the ocean have been reported in previous literature (*28*, *29*), those ideas cannot be directly applied to West Antarctica due to the lack of ice-ocean interactions and the prominent differences in hydrographic conditions, topographic geometry, and wind forcing. Thus, the mechanism underlying the formation of the West Antarctic Slope Undercurrent is still ambiguous.

In this study, the dynamics of the West Antarctic Slope Undercurrent are investigated using a high-resolution process model with coupled ocean, sea ice, and ice shelf components. We show that there is a positive feedback between the undercurrent and ice shelf melt. Stronger meltwater upwelling leads to stronger slope undercurrent and enhanced onshore heat transport in a warming climate, accelerating future melt of ice shelves. We reveal the mechanism of undercurrent formation and provide a mechanistic explanation of this positive feedback by analyzing the vorticity budget within the CDW layer. The theory is further verified by a suite of sensitivity

[1]Department of Atmospheric and Oceanic Sciences, University of California, Los Angeles, Los Angeles, CA, USA. [2]Ocean and Earth Science, National Oceanography Centre, University of Southampton, Southampton, UK.
*Corresponding author. Email: y_si@mit.edu
†Present address: Department of Earth, Atmospheric, and Planetary Sciences, Massachusetts Institute of Technology, Cambridge, MA, USA.

experiments with varying winds, tides, diapycnal mixing, and geometry.

RESULTS

Simulating West Antarctic Slope Undercurrent formation and ice shelf melt

Figure 1 illustrates the model configuration. An ocean general circulation model with coupled ocean, sea ice, and ice shelf components has been configured into a 600 km (zonal) × 400 km (meridional) × 4000 m (vertical) domain, with the ice shelf front and coastline at latitudinal distances of 100 and 120 km from the southern boundary, respectively. The model bathymetry is inspired by the typical ocean bathymetry in the Amundsen Sea, West Antarctica (*30*), where the slope undercurrent has been observed. The continental shelf has a depth of 500 m at the shelf break, deepening toward the southern boundary with a 300-m change in bedrock elevation (H_{bed} = 300 m, Fig. 1A). There is a submarine trough that connects the shelf break to the ice shelf cavity, with a depth of 300 m and a width of 30 km. The model is forced by time-invariant surface air conditions that are representative of the annual-mean state of the Amundsen Sea, with steady northwestward winds weakening linearly offshore (Fig. 1A and Materials and Methods). We restore ocean temperature, salinity, velocity, and sea ice properties including sea ice thickness, concentration, and sea ice velocity at the zonal boundaries and northern boundary within a 20-km-wide sponge layer (Materials and Methods). Note that boundary zonal velocities are directed westward (Fig. 1B), i.e., the formation of the eastward undercurrent is not imposed by the boundary conditions. Further details on model configuration, including boundary conditions, surface air conditions, model resolution, viscosity, diffusivity, tides, etc., are provided in Materials and Methods.

As listed in table S1, we conducted perturbation experiments with varied winds, tides, vertical diffusivity, change in bedrock elevation on the continental shelf (H_{bed}), and width of the trough (W_{tr}). We additionally ran simulations with different boundary conditions (a deeper thermocline at all boundaries) or different shelf geometry (no trough over the shelf). We covary thermocline depth, winds, and shelf geometry in some simulations. We choose to vary these parameters because: (i) winds and tides are the primary mechanical forcings of the ice-ocean system in the Antarctic Slope Current (*31*); (ii) observations and numerical models have shown that the basal melt rate is correlated with the thickness of CDW over the shelf (*20*, *32*), which is set by the difference between the thermocline depth and bedrock elevation; (iii) submarine troughs affect the speed of the undercurrent (*10*) and shelf/slope exchange (*33*); and (iv) vertical mixing modulates isopycnal geometry, possibly affecting the strength of the undercurrent. To explicitly investigate how meltwater affects the shelf/slope circulation and on-shelf heat transport, we designed four simulations with a pseudo-ice shelf, in which the ice shelf thermodynamics were turned off and meltwater fluxes were prescribed at the tilted interface between the ice shelf and the ocean (Materials and Methods).

The reference simulation captures the salient features of the circulation system, heat transport, and melt rate in the Amundsen Sea. The sea surface height increases toward the continental shelf (fig. S1,

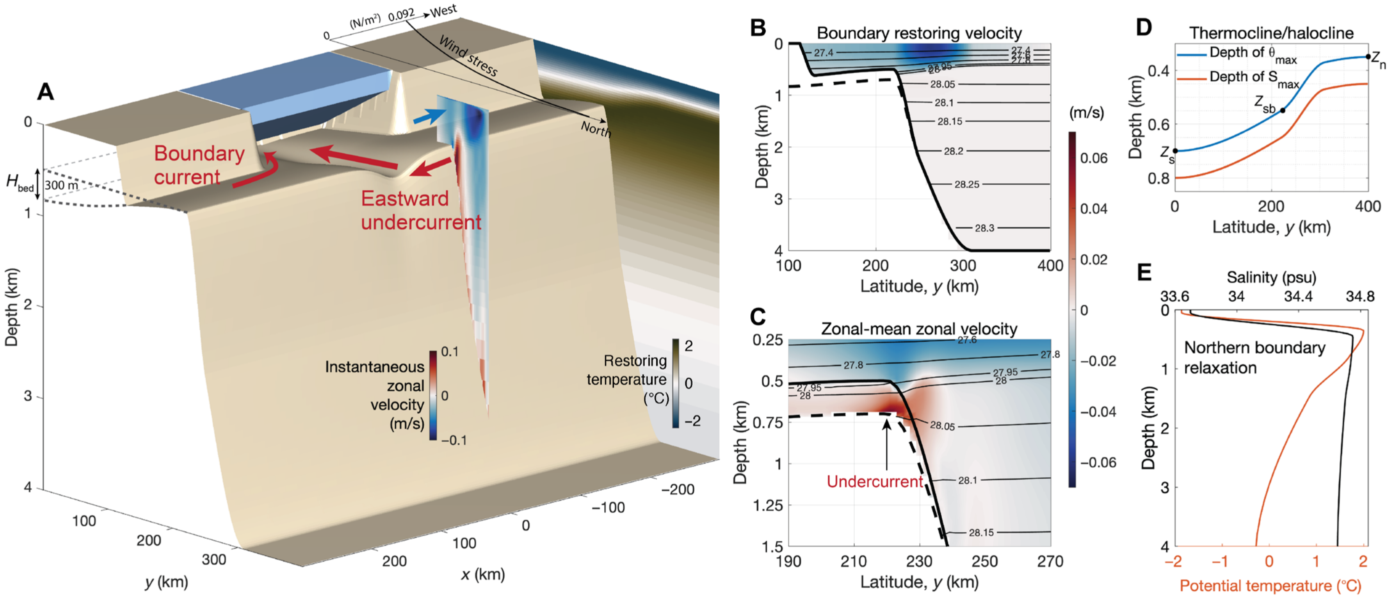

Fig. 1. Model configuration. (**A**) Model bathymetry, ice shelf, wind stress amplitude, restoring temperature at the zonal boundaries, and a snapshot of zonal velocity over the continental slope. (**B**) Zonal velocity (color) and neutral density (thin black contours) prescribed at the two sponge layers of the eastern and western boundaries. The boundary-layer zonal velocity is set by vertically integrating the thermal wind relation from the seafloor to level *z*, assuming zero velocity at the seafloor. The shallowest and deepest bathymetric contours are indicated by the thick solid and dashed lines, respectively. (**C**) Zonal-mean zonal velocity overlaid by neutral density contours in the reference simulation. The 20-km zonal sponge layers at the zonal boundaries have been excluded from the calculation of the zonal mean. (**D**) Depth of the maximum potential temperature and salinity restored at the zonal boundaries, which is also prescribed as an initial condition across the domain. For a stable stratification, the salinity maximum needs to be deeper than the temperature maximum. The depth of maximum potential temperature at the northern boundary (Z_n) is fixed to 350 m for all simulations. The depth of maximum potential temperature at the southern boundary (Z_s) and the shelf break (Z_{sb}) of the zonal boundaries are varied in some simulations (table S1). (**E**) Northern boundary restoring temperature and salinity.

A and D) due to wind-induced Ekman transport and the imposed inflow boundary conditions with a westward geostrophic flow. The CDW (defined as potential temperature warmer than 0°C hereafter) layer thickness is about 250 m over the continental shelf and 550 m in the trough, increasing to about 2.6 km in the deep ocean (fig. S1, B, E, and H). Consistent with Jacobs *et al.* (*5*), the CDW potential temperature reaches 1.5°C over the shelf and slope, nearly 3.5°C above the freezing temperature (fig. S1, C, F, and I). The circulation in the CDW layer is cyclonic in the trough, consisting of a narrow, eastward slope undercurrent upstream (west) of the trough, a strong southward CDW inflow, and a northward return flow (Fig. 2A). Figure S2 shows the cross sections of potential temperature, salinity, and zonal velocity in the reference simulation, which agree well with the observations of Amundsen Sea Slope Undercurrent by Walker *et al.* (*10*). The undercurrent is stronger upstream of the trough and substantially weakened downstream of the trough, suggesting a net onshore flow within the trough. Both in nature and in the simulations, the eastward undercurrent is present despite the strong mechanical forcing due to winds and tides that favors westward flow. In addition, the undercurrent transports sufficient heat through the trough to the ice shelf to cause a realistic amount of basal melt (16.0 m/year) in the reference simulation, which is comparable with the melt rates of major ice shelves in the Amundsen Sea [e.g., 16.2 ± 1 m/year for Pine Island and 17.7 ± 1 m/year for Thwaites in (*3*); 14.0 ± 1.6 m/year for Pine Island in (*34*)].

Buoyancy-driven slope undercurrent and onshore CDW heat transport

The perturbation simulations indicate a correlation between the undercurrent transport (Eq. 16) and ice shelf melt (Fig. 3E and fig. S4C). The undercurrent is relatively strongly modulated by processes that change the isopycnal geometry, such as tides, buoyancy forcing, and diapycnal mixing. The ice shelf melt rate is insensitive to variations in local wind speed, which might be an artifact resulting from the zonal boundary conditions in our model. Details on the melt rate sensitivity are discussed in the "Ice shelf melt rate sensitivity" section in Materials and Methods.

Since in our modeling framework winds and tides exert forces that oppose the undercurrent, the correlation between undercurrent and ice shelf melt raises the hypothesis that the undercurrent is driven by sub-ice shelf buoyancy fluxes. To evaluate this hypothesis and provide insight into how basal melt influences shelf/slope circulation and shoreward heat transport, we designed four controlled experiments with a "pseudo-ice shelf." In these experiments, we turn off ice shelf thermodynamics and prescribe heat and salt fluxes at the tilted interface between the pseudo-ice shelf and the ocean (see Materials and Methods) to create equivalent melt rates of $\omega_{melt} = 0$, 8, 16, and 24 m/year. Figure 4 shows that increased meltwater flux drives stronger shoreward CDW transport in the trough (-50 km $\le x \le 50$ km, 100 km $\le y \le 225$ km), increasing from 0.015 Sverdrup (Sv) (no melt, Fig. 4A; 1 Sv = 10^6 m^3/s) to 0.64 Sv (strong melt, Fig. 4G). To quantify where CDW enters the ice shelf cavity, we calculate the cumulative CDW heat transport integrated westward along the ice shelf front, as a function of longitude. When the ice shelf is not melting, the cumulative CDW heat transport is zero at the ice front (Fig. 4, A and B); with stronger prescribed meltwater flux, there is increased total CDW heat transport toward the ice shelf (Fig. 4, C to H). Note that shoreward transport of CDW in the trough is not a guaranteed outcome in these experiments. The system could, in principle, have instead adjusted to a state in which surface waters are lightened/cooled in the cavity by the imposed buoyancy fluxes and then exported offshore, or in which CDW is drawn in along the coast from the zonal domain boundaries.

In addition, the eastward transport of the slope undercurrent doubles with a strong prescribed melt rate ($\omega_{melt} = 24$ m/year) compared with no melt ($\omega_{melt} = 0$ m/year). A weak undercurrent appears in the simulation with no melt (Fig. 4A), provided that there is an offshore buoyancy gradient and a trough over the continental slope, the mechanism of which is discussed in the "Coriolis term and diapycnal upwelling" section in Materials and Methods. Note that this does not conflict with the hypothesis that the undercurrent is buoyancy-driven because, in the real ocean, the offshore buoyancy gradient is established by meltwater from local ice shelves as well as remote sources. In agreement with Thompson *et al.* (*35*) and Flexas *et al.* (*36*), our results emphasize the potential control by remote buoyancy forcing (e.g., meltwater advection from upstream ice shelves) on shoreward heat transport in West Antarctica.

In the rest of this section, we investigate the formation mechanism of the buoyancy-driven slope undercurrent. Since the slope undercurrent and the shoreward CDW transport in the trough form a cyclonic circulation (Fig. 2A), the question of what drives the undercurrent can be narrowed down to the following: (i) Which process injects cyclonic vorticity into the system? (ii) How is the cyclonic vorticity transported to the shelf break? This motivates us to analyze the vorticity budget of the CDW layer.

By calculating the curl of the depth-integrated CDW momentum equation in a steady state (see Materials and Methods), it is shown that meltwater upwelling in the ice shelf cavity injects cyclonic vorticity into the CDW layer. Figure 5 shows the vorticity budget of the CDW layer, with blue corresponding to the contribution of each term to cyclonic vorticity ($\zeta < 0$), and red anticyclonic ($\zeta > 0$). For the leading-order terms, the total advection is balanced by total pressure torque; the dissipation is nonnegligible only over the shelf break. As CDW melts the ice shelf, the meltwater transforms CDW into a lighter, fresher water mass, which corresponds to a diapycnal velocity out of the CDW layer. In the ice shelf cavity, the Coriolis term, which is associated with diapycnal upwelling, contributes to the cyclonic vorticity in the CDW layer (Fig. 5G and Eq. 15). The total pressure torque of the CDW layer is composed of the bottom pressure torque (BPT) exerted by the titled topography onto the flow and the interfacial pressure torque (IPT) exerted by the titled isotherm between the CDW layer and the surface layer. As CDW flows from the shelf break to the ice front in the eastern part of the trough, the downward bottom velocity associated with the deepening of the topography leads to negative BPT (Fig. 5D, fig. S3A, and Eq. 12a), which is partially canceled by the IPT due to the downward velocity at the top of the CDW layer (Fig. 5E, fig. S3B, and Eq. 12b). Vertical stretching of the CDW layer in the eastern part of the trough is associated with the negative total pressure torque (Fig. 5A, fig. S3C, and Eq. 13). Other terms such as vorticity advection, vertical advection, and kinetic energy gradient are less important.

On the basis of Stokes' theorem, the area integral of vorticity over an enclosed surface is equal to the line integral of circulation around the boundary of the surface. To explain what drives the cyclonic circulation of the undercurrent, we calculate the area integral of the vorticity budget. We use PV contours to select a region (Fig. 2B), because a flow that is close to adiabatic and frictionless approximately follows PV contours. The PV of the CDW layer is defined as

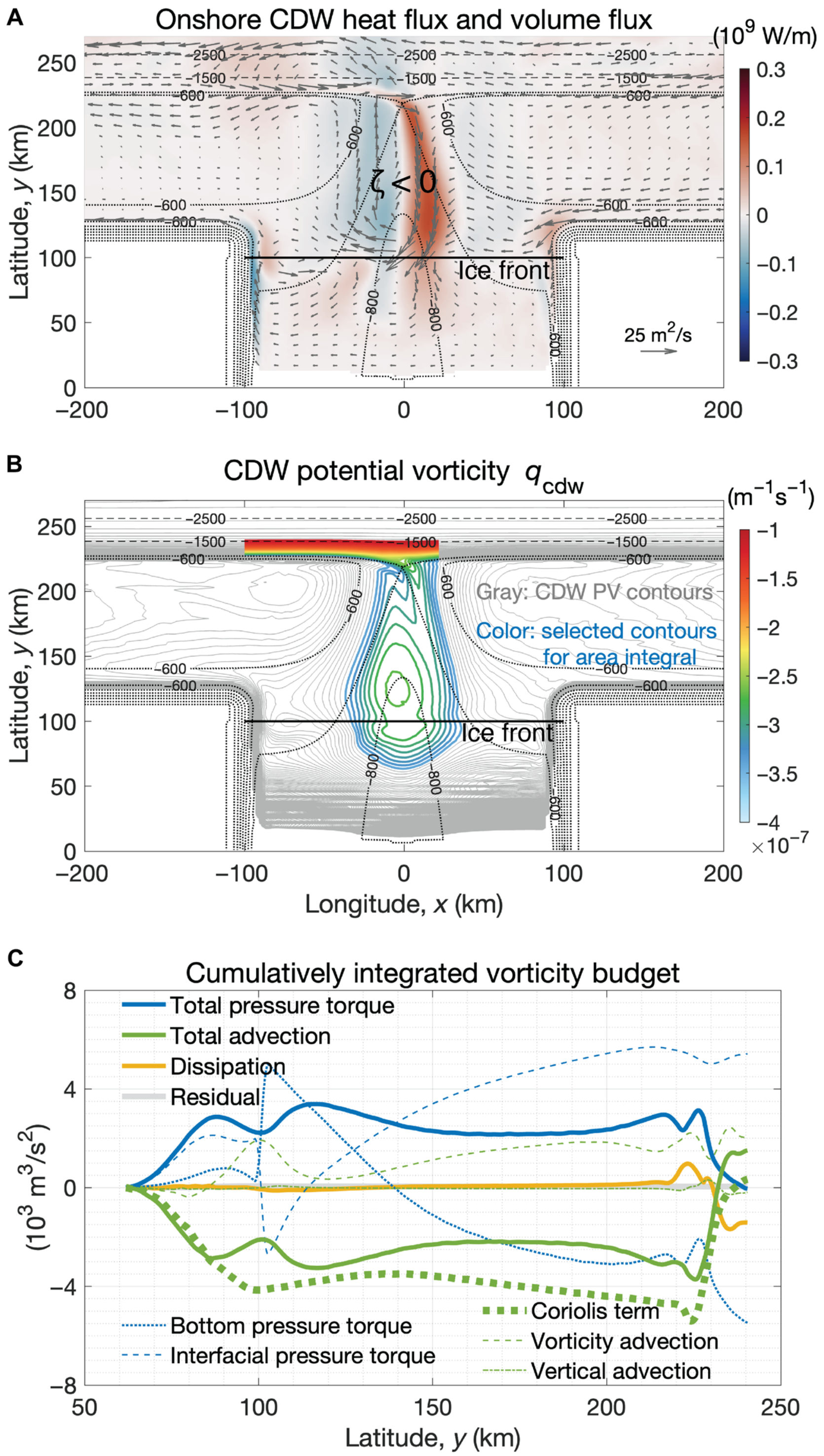

Fig. 2. Area-integrated vorticity budget of the cyclonic circulation in the CDW layer. (**A**) Onshore CDW heat flux (color) and CDW volume flux (arrows) in the reference simulation, with red corresponding to shoreward (southward) heat flux. There is a cyclonic flow (relative vorticity $\zeta < 0$) comprising the slope undercurrent and the CDW circulation in the trough. The black solid line denotes the location of the ice shelf front at $y = 100$ km. The bathymetric contours are denoted by the thin dashed lines with an interval of 1000 m and thin dotted lines with an interval of 100 m. (**B**) PV of the CDW layer denoted by the gray contours with an interval of 10^{-8} m^{-1} s^{-1}. The colored PV contours denote the selected region based on the CDW volume flux shown in (A), with the maximum and minimum PV values of -1.0×10^{-7} and -3.5×10^{-7} m^{-1} s^{-1}, respectively. Over the shelf break and upper slope (225 km $\le y \le$ 240 km), the selected region is cut off at longitudes $x = -100$ km and $x = 40$ km, respectively. (**C**) The vorticity budget of the CDW layer cumulatively integrated from south to north within the selected region shown in (B), as a function of latitude. The cumulatively integrated vorticity budget is insensitive to the cutoff longitude or the selected PV values provided that the pathway of the CDW over the continental shelf is mostly included in the selected region.

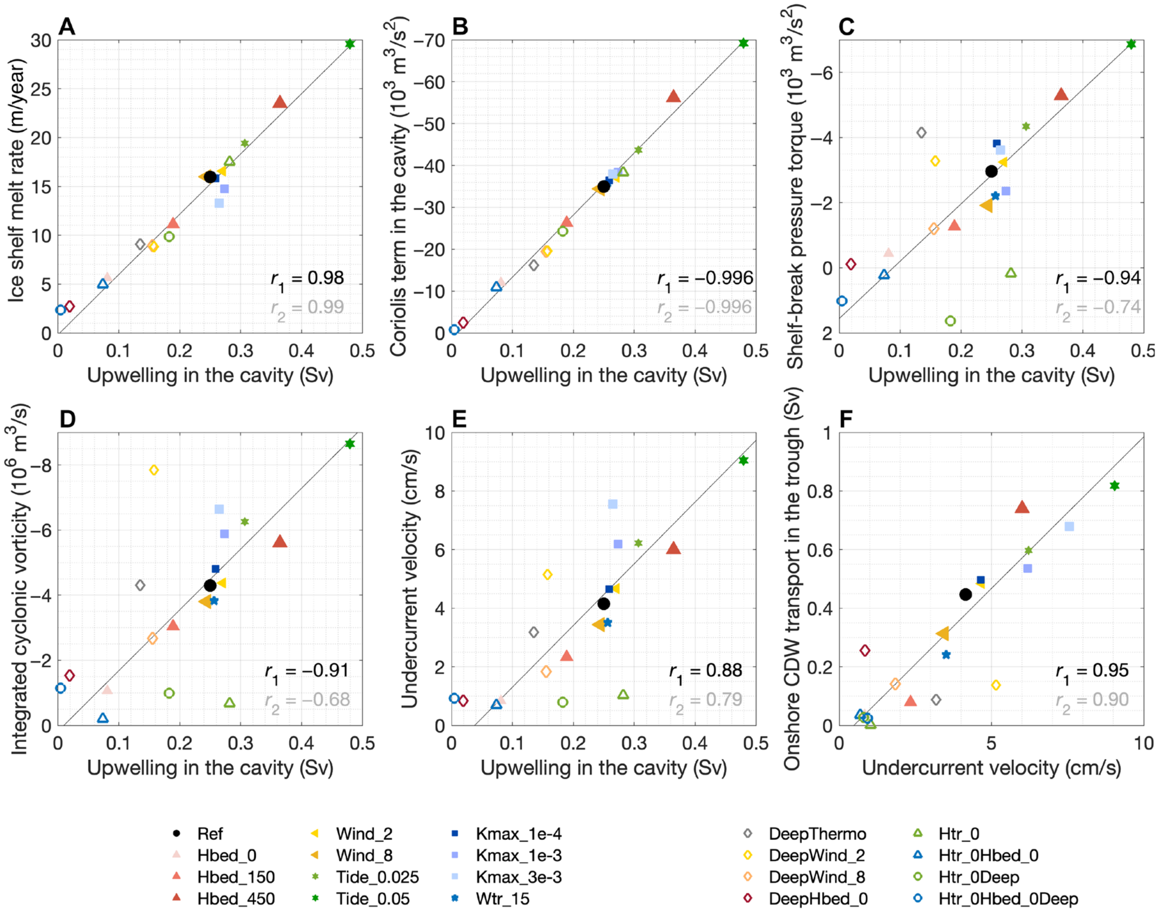

Fig. 3. Perturbation experiments. Correlation between the diapycnal upwelling across the 0°C isotherm in the ice shelf cavity with (**A**) ice shelf melt rate, (**B**) Coriolis term in the vorticity budget of the CDW layer integrated within the cavity, (**C**) total pressure torque of the CDW layer integrated over the shelf break west (upstream) of the trough (210 km ≤ y ≤ 235 km, −120 km ≤ x ≤ 0 km, shown by the green box in Fig. 5J), (**D**) cyclonic vorticity integrated over the shelf break west of the trough, and (**E**) transport-weighted undercurrent velocity west of the trough (Eq. 17). (**F**) Correlation between transport-weighted undercurrent velocity and shoreward CDW transport within the trough (1 Sv = 10^6 m/s; Eq. 19). In each panel, r_1 is the correlation coefficient for simulations with fixed topographic geometry and boundary isopycnal geometry, denoted by the filled markers; r_2 is the correlation coefficient for all simulations, including simulations with varied topographic geometry and boundary isopycnal geometry, denoted by the hollow markers; the black thin line is the linear fit for the filled markers. The names of the experiments in the figure are consistent with table S1.

$$q_{cdw} \equiv \frac{f + \partial_x v_{cdw} - \partial_y u_{cdw}}{h_{cdw}} \qquad (1)$$

where f is the Coriolis parameter, u_{cdw} and v_{cdw} are the depth-averaged zonal and meridional velocities of the CDW layer, and h_{cdw} is the CDW thickness. Figure 2C shows the cumulatively integrated vorticity budget from south to north within the selected region as a function of latitude. As in Fig. 5G, Fig. 2C shows that the Coriolis term associated with meltwater upwelling in the ice shelf cavity injects cyclonic (negative) vorticity into the system. Furthermore, the area-integrated total pressure torque is zero (the value at y = 240 km in Fig. 2C), suggesting that the pressure torque is neither a source nor a sink of the CDW vorticity; instead, it acts to horizontally transport the cyclonic vorticity from the ice shelf cavity to the shelf break (*37*) (Eq. 14 in Materials and Methods).

Using a suite of perturbation experiments, we consolidate the theory that as CDW melts the ice shelf, diapycnal upwelling of meltwater generates cyclonic vorticity, which is transported from the ice shelf cavity to the shelf break by the vorticity flux associated with the total pressure torque (referred to as "pressure-torque vorticity flux" hereafter, Eq. 14), leading to steering of the undercurrent toward the ice shelf. It is verified that higher ice shelf melt rates lead to stronger diapycnal upwelling in the cavity (Fig. 3A) and enhanced cyclonic vorticity input to the CDW layer by the Coriolis term (Fig. 3B). For simulations with fixed topographic geometry and boundary isopycnal (filled markers with correlation coefficient r_1 in each panel), upwelling in the cavity is highly correlated with the total pressure torque and cyclonic vorticity integrated over the shelf break upstream of the trough (defined by the green box in Fig. 5J) (Fig. 3C, D). As a result, stronger ice shelf melt enhances the eastward transport of the slope undercurrent (Fig. 3E), which drives stronger shoreward CDW transport within the trough (Fig. 3F) and thus a larger magnitude of heat reaching the ice shelf front (fig. S4A). Further discussion of the perturbation experiments is included in the "Pressure torques and vertical stretching of the CDW layer" section in Materials and Methods.

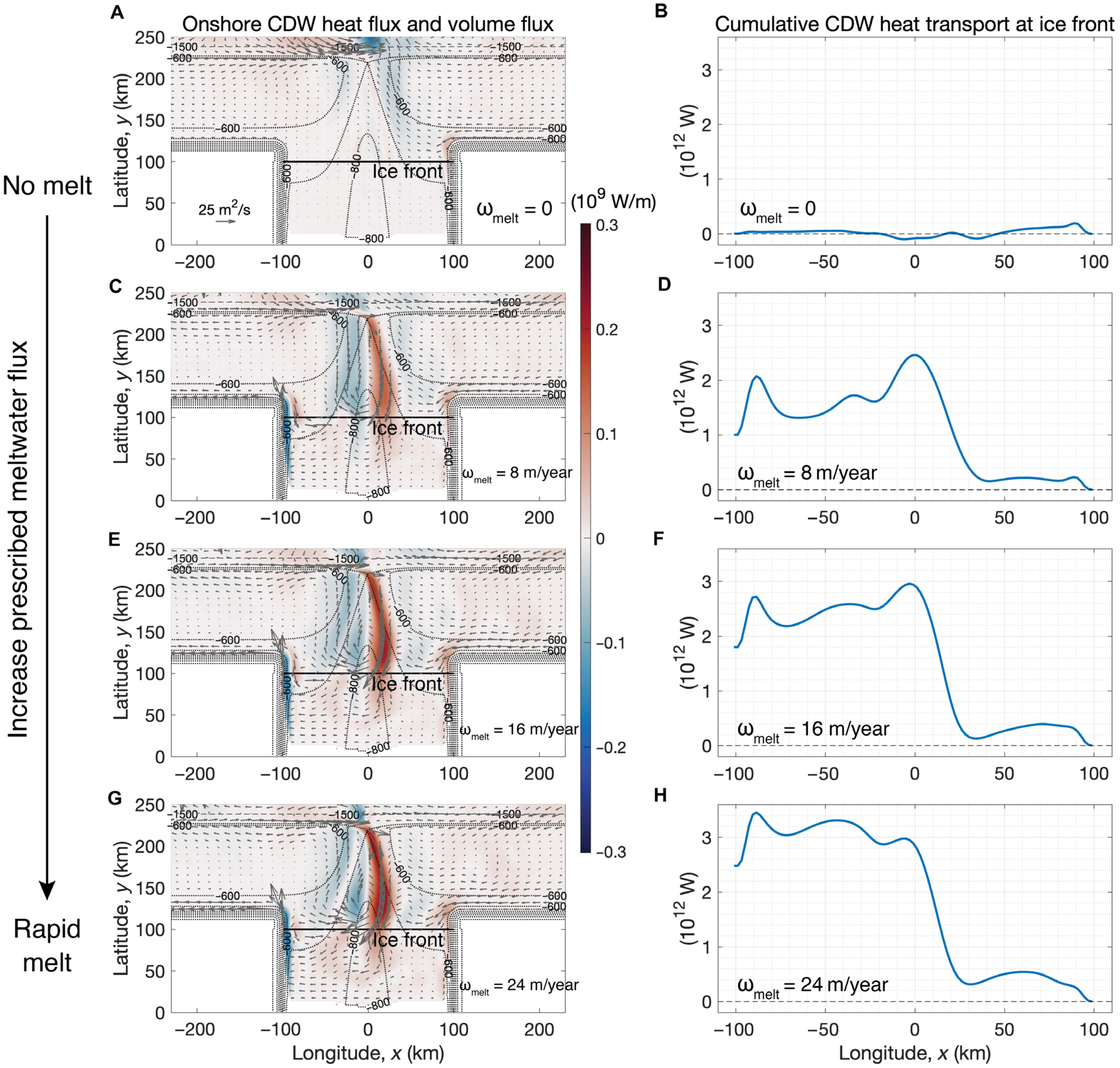

Fig. 4. Onshore heat transport in the CDW layer increases with a larger prescribed meltwater flux. Left column: Vertically integrated heat flux (color) and volume flux (arrows) in the CDW layer, with red corresponding to shoreward (southward) heat flux. The black solid line denotes the location of the ice shelf front at $y = 100$ km. The bathymetric contours are denoted by the thin dashed lines with an interval of 1000 m and thin dotted lines with an interval of 100 m. Right column: Cumulative CDW heat transport at the ice shelf front as a function of longitude. The CDW heat flux is first integrated vertically and then integrated zonally along the ice shelf front, from $x = 100$ km to $x = -100$ km. (**A** and **B**) Pseudo-ice shelf with no melt. (**C** to **H**) The equivalent basal melt rates prescribed at the titled surface of the pseudo-ice shelf (ω_{melt}) are 8, 16, and 24 m/year, respectively. Configurations of the pseudo-ice shelf simulations are presented in the "Pseudo-ice shelf" section in Materials and Methods and in table S1.

DISCUSSION

Implications for future melt of West Antarctic ice shelves

In this study, the mechanism of the Antarctic Slope Undercurrent formation is investigated using an idealized model with coupled ocean, sea ice, and ice shelf components. It is found that the undercurrent forms with realistic strength provided that there is a trough allowing access to the continental shelf and ice shelf cavity and that there is a cross-slope buoyancy gradient. The cross-shelf buoyancy gradient is mainly contributed by ice shelf meltwater (*38*) and is maintained by wind-driven shoreward Ekman transport of fresh surface waters. The controlled experiments with the pseudo-ice shelf demonstrate that the shoreward heat transport in the trough is

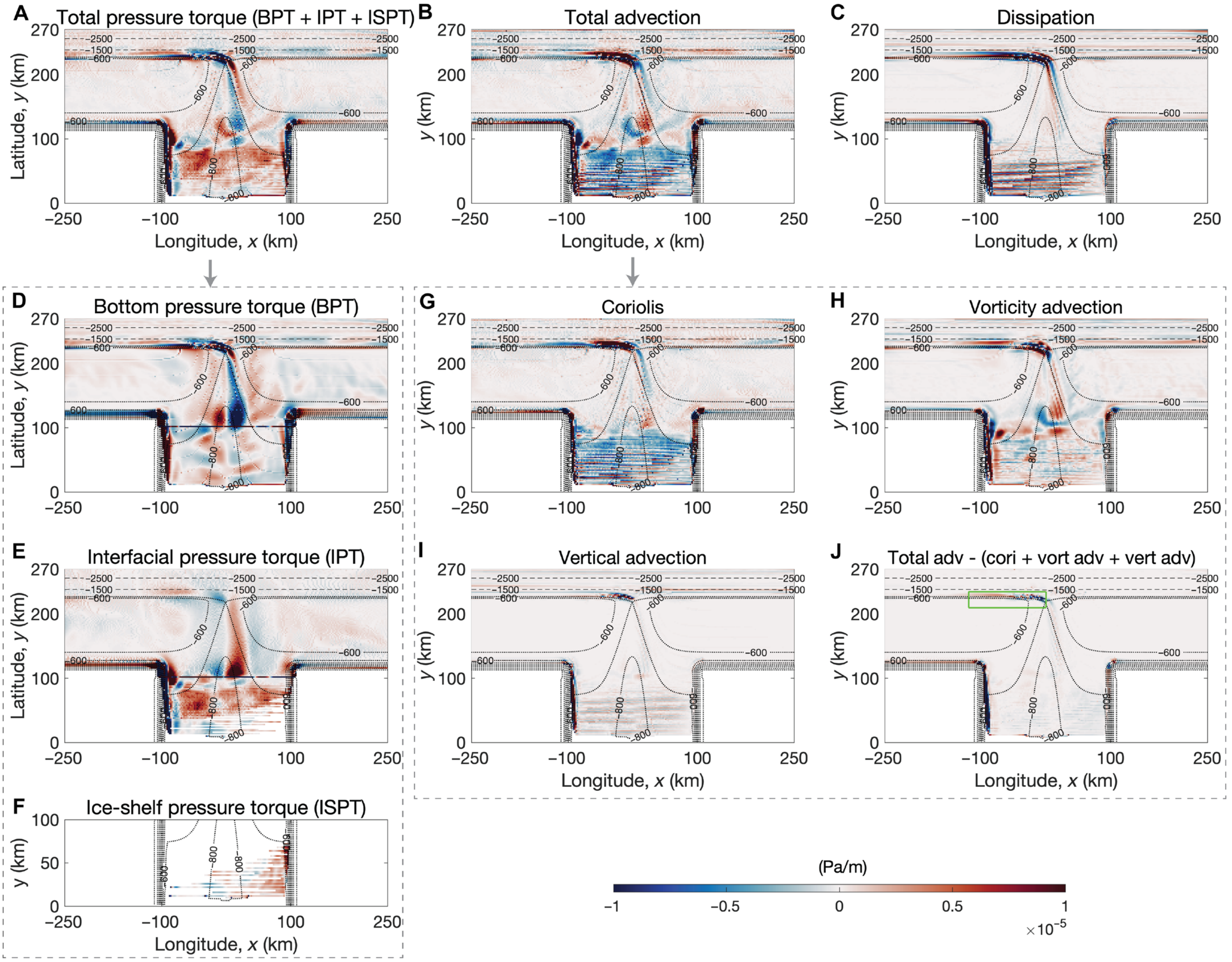

Fig. 5. Vorticity budget of the CDW layer. The total pressure torque (**A**) balances total advection (**B**) and dissipation (**C**). The total pressure torque of the CDW layer comprises the BPT (**D**), IPT (**E**), and ice shelf pressure torque (**F**). The total advection comprises the Coriolis term (**G**), vorticity advection (**H**), vertical advection (**I**), and the residual term (**J**). The bathymetric contours are denoted by the thin dashed lines with an interval of 1000 m and thin dotted lines with an interval of 100 m. The green box in (J) indicates the selected region to calculate the shelf-break–integrated relative vorticity and pressure torque of the CDW layer, presented in Fig. 3 (C and D) and fig. S4D, respectively.

driven by sub-ice shelf buoyancy fluxes. We establish a direct dynamical link between the undercurrent transport and ice shelf melt using the vorticity balance within the layer of intruding CDW. Our results suggest that the Antarctic Slope Undercurrent observed in West Antarctica is at least partially driven by cyclonic vorticity input from meltwater upwelling in the ice shelf cavities. Figure 6 illustrates the mechanism of Antarctic Slope Undercurrent formation. As CDW melts the ice shelf, diapycnal upwelling of the meltwater vertically stretches the CDW layer, injecting cyclonic vorticity into that layer. The cyclonic vorticity is transported from the ice shelf cavity to the shelf break by the vorticity flux associated with the total pressure torque, which drives the slope undercurrent and leads to steering of CDW toward the ice shelf. The undercurrent forms down-wave (in the direction of topographic Rossby wave propagation) (*39*) of the buoyancy forcing, which is consistent with the above interpretation of the undercurrent as a buoyancy-driven circulation. Increased basal melt drives a stronger slope undercurrent and enhanced onshore CDW heat transport, which indicates a positive feedback that may accelerate future melt of ice shelves, potentially further destabilizing the West Antarctic Ice Sheet.

We focus on the steady state of the West Antarctic Slope Undercurrent and not on fluctuations, because regional models show that the undercurrent is always present, regardless of season or year [e.g., (*13*)]. For the annual-mean state, sea ice formation leads to buoyancy loss over the continental shelf (*38*), so it is not able to explain the emergence of the undercurrent or the slope front structure of the Amundsen Sea. If winter mixing intrudes deeply enough to entrain the CDW layer and influences its vorticity budget, then the strong seasonally varying surface buoyancy forcing from sea ice could possibly overwhelm the influence of ice shelf meltwater on

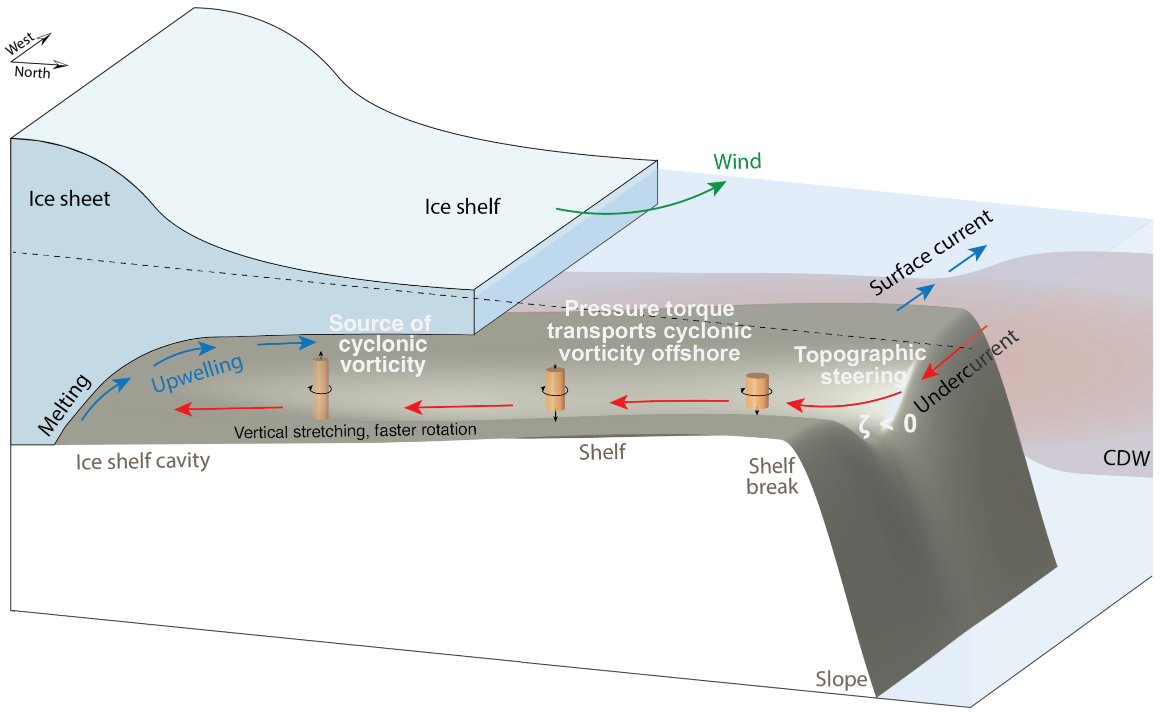

Fig. 6. Schematic illustrating the mechanism of Antarctic Slope Undercurrent formation. The ice shelf meltwater creates an offshore buoyancy gradient with shoreward deepening of the thermocline, which is maintained by shoreward Ekman transport associated with westward coastal winds. As CDW melts the ice shelf, meltwater transforms the CDW into a lighter water mass and vertically stretches the CDW layer, injecting cyclonic vorticity into the CDW layer. The source of the cyclonic vorticity is then transported offshore by the pressure-torque vorticity flux (Eq. 14), provided that there is a trough extending onto the shelf. Over the shelf break, the cyclonic vorticity steers CDW toward the ice shelf, driving an eastward undercurrent west of the trough. Stronger basal melt leads to stronger diapycnal upwelling of meltwater, increasing the strength of the slope undercurrent and therefore enhancing onshore CDW heat transport. The vertical stretching of the CDW layer is illustrated by the orange columns, with black arrows at the top or bottom of the columns denoting the direction of the stretching and circular arrows denoting the resulting cyclonic rotation. As CDW flows toward the ice shelf, it is primarily stretched downward following the topography (fig. S3); within the ice shelf cavity, the CDW layer is stretched upward due to the upwelling of meltwater.

shorter timescales [figure 8A in (*38*)]. Lacking seasonality is a caveat of this work that warrants future study. Since remote and local buoyancy forcings can also affect the offshore buoyancy gradient and thus undercurrent strength, our results emphasize their control of shoreward heat transport in West Antarctica. In a warming climate, if increased freshwater input (e.g., from icebergs, sea ice loss, and upstream ice shelves) leads to changes in the slope front structure, then it might result in changes in the basal melt, as this would influence the establishment of the shelf-break BPT and the strength of the undercurrent.

Our idealized model does not account for various complexities such as ice shelf morphology (*23*), the presence of multiple troughs along the West Antarctic continental slope, the occurrence of multiple ice shelves, and the wide range of forcing timescales. In addition, the continental shelf of our model is relatively narrow compared to the Amundsen Sea shelf. Unlike previous studies [e.g., (*13*, *16*, *21*)], the ice shelf melt rate is insensitive to wind stress variations, which may be a model artifact due to the zonal boundary conditions (see the "Ice shelf melt rate sensitivity" section in Materials and Methods). Our idealized model ensures a zonal import of CDW from the eastern boundary, which may not exist in the real world. In the real Amundsen Sea, a ridge blocks westward CDW inflow from the Bellingshausen Sea [e.g., figure 3B in (*40*)]. Using an idealized ocean model, Haigh *et al.* (*40*) found that the addition of a meridional ridge east of the ice shelf results in a deep cyclonic circulation over the shelf and induces an undercurrent along the shelf break, but their model does not include any thermodynamic forcing from sea ice and ice shelf and therefore lacks potentially important feedbacks between ocean circulation and ice shelf melt. As revealed by St-Laurent *et al.* (*41*), the interaction of the mean flow and Rossby waves with the topography plays a role in driving southward heat transport through a coastal trough. How complex topography, high-frequency wind variability, transient eddies, and waves affect the dynamics of the undercurrent and shoreward heat transport in West Antarctica requires further study.

Our results imply that models without ice shelf cavities are likely to miss an important positive feedback between buoyancy-driven onshore heat transport and ice shelf basal melt, which should be taken into consideration in future modeling studies on the Antarctic continental shelves. Subsequent investigations and assessments of sea level rise scenarios should focus on the positive feedback demonstrated here as a potentially important player in the future evolution and stability of the West Antarctic Ice Sheet.

MATERIALS AND METHODS

Model configuration

The model is developed on the basis of the Massachusetts Institute of Technology General Circulation Model (MITgcm) (*42*, *43*). The

ocean component is modeled via the hydrostatic Boussinesq equations with a polynomial approximation of the equation of state (*44*). A fully dynamic-thermodynamic sea ice package is implemented in the model, with viscous-plastic sea ice rheology and seven thickness categories for sea ice thermodynamics (*45*–*48*). The model simulates thermodynamic ice-ocean interactions at the base of a static ice shelf using friction velocity-dependent turbulent heat and salt transfer coefficients (*49*) and also includes the pressure and quadratic frictional stress of the ice shelf onto the waters beneath. The ice shelf thickness decreases linearly from 800 m at the grounding line at the southern boundary to 200 m at the ice shelf front, with a meridional extent of 100 km and a zonal extent of 200 km (Fig. 1A). The density and specific heat capacity of the ice shelf are 917 kg/m^3 and 2000 J/kg per K, respectively. The surface temperature on the top of the ice shelf is −20°C.

The baroclinic Rossby radius of deformation is about 3.9 km over the shelf and 9.8 km in the deep ocean. Therefore, a high horizontal resolution is required to resolve mesoscale eddies over the shelf, which limits the domain size. The horizontal resolution of the model is 2 km, and the vertical grid spacing increases from 3.3 m near the ocean surface to 333 m toward the seafloor, with 68 vertical layers in total. The vertical grid spacing in the ice shelf cavity ranges from 25 to 29 m. Although the vertical resolution is known to have an influence on the representation of ice shelf melt rates (*50*), increasing the number of vertical layers from 68 to 100 leads to only a 3% decrease in the melt rates in this model; therefore, we use 68 vertical levels for all the simulations.

A high-order nonlinear advection scheme (seventh-order one-step method with monotonicity-preserving limiter) is implemented for potential temperature, salinity, and sea ice scalar state variables. Standard bulk formulae are used to compute the atmosphere-sea ice-ocean fluxes of momentum, heat, and freshwater. For both ocean and sea ice, the inner and outer relaxation timescales are 10 and 0.5 days, respectively. The time step is approximately 170 s.

The boundary hydrography is inspired by observations (*5*, *51*) with a mixed-layer depth of approximately 50 m and a thermocline depth of approximately 300 m at the northern boundary (Fig. 1E). The boundary conditions at the two zonal boundaries are the same, with the thermocline and halocline deepening toward the coastline (Fig. 1D). The depth of the salinity maximum is prescribed to be 100 m deeper than the potential temperature maximum at the zonal boundaries, to obtain a stable stratification. At the northern boundary, both the zonal and meridional velocities are restored to zero except for two simulations with prescribed barotropic tides in the meridional direction. Note that the reference simulation does not include tides. At the zonal boundaries, the zonal velocity is prescribed by assuming zero seafloor velocity and vertically integrating the thermal wind shear from the seafloor to depth z (Fig. 1B)

$$u_{\text{boundary}}(y,z) = \int_{\eta_b}^{z} \frac{g}{\rho_0 f} \frac{\partial \rho_{\text{boundary}}(y,z)}{\partial y} dz' \quad (2)$$

where η_b is the seafloor elevation, g is the gravitational acceleration, ρ_0 is the reference density, f is the Coriolis parameter, and ρ_{boundary} is the potential density calculated from the restoring temperature and salinity at the zonal boundaries.

In our simulations, a coastal polynya is formed in front of the ice shelf, which resembles the Amundsen Sea Polynya. Because of sea ice production in coastal polynyas, sea ice inputs negative buoyancy forcing into the Amundsen Sea (*38*, *52*), which is about three times smaller than the positive buoyancy forcing from ice shelf melting [figure 5 in (*38*)] in terms of annual mean. In the model, the following parameters of surface air conditions are selected to represent the annual-mean state of the Amundsen Sea and to ensure that the sea ice thickness is approximately constant over the course of the simulation, so that sea ice buoyancy forcing is much smaller compared with sub-ice shelf buoyancy forcing: the surface (2 m) air temperature is −10°C, the surface specific humidity is 5.7 g/kg, the downward longwave radiation is 324 W/m^2, and the downward shortwave radiation is zero [the same as (*53*)]. The input of fresh water from precipitation was neglected.

Assuming a free drift sea ice boundary condition, we solve Eqs. 3a and 3b for sea ice velocities (u_i, v_i) for each latitude at the zonal boundaries. The Coriolis term on the left-hand side is balanced by the air-ice stress and ice-ocean stress on the right-hand side

$$-\rho_i h_{i0} f v_i = \rho_a C_{ai} \sqrt{u_a^2 + v_a^2}\, u_a - \rho_o C_{io} \sqrt{(u_i - u_o^s)^2 + v_i^2}\, (u_i - u_o^s) \quad (3a)$$

$$\rho_i h_{i0} f u_i = \rho_a C_{ai} \sqrt{u_a^2 + v_a^2}\, v_a - \rho_o C_{io} \sqrt{(u_i - u_o^s)^2 + v_i^2}\, v_i \quad (3b)$$

where ρ_i = 920 kg/m^3 is the sea ice density, ρ_a = 1.3 kg/m^3 is the air density, ρ_o = 1027 kg/m^3 is the reference density of the seawater, h_{i0} = 1 m is the boundary sea ice thickness, $C_{ai} = 2.0 \times 10^{-3}$ is the air-ice drag coefficient, $C_{io} = 5.54 \times 10^{-3}$ is the ocean-sea ice drag coefficient, (u_a, v_a) are the wind velocities as a function of latitude, and $u_o^s = u_{\text{boundary}}(y, 0)$ is the ocean surface zonal velocity at the zonal boundaries. We conducted simulations with varying meridional and zonal winds (table S1), with stronger winds corresponding to increased sea ice velocity and larger ice-ocean shear. Other parameters of sea ice and drag coefficients are the same as (*53*): The sea ice salinity retention fraction on freezing is 0.3; the frazil to sea ice conversion rate is 0.01; the quadratic air-ocean drag coefficient is 1×10^{-3}; the quadratic ocean bottom drag coefficient is 2×10^{-3}.

The model is implemented with the Smagorinsky viscosity with a biharmonic viscosity factor of 4 (*54*–*56*). The vertical eddy viscosity is set to 3×10^{-4} m^2 s^{-1}. For most simulations, a uniform vertical diffusivity of 10^{-5} m^2/s is implemented. In three perturbation simulations, we allow three-dimensional (3D) specification of vertical diffusivity. The 3D diffusivity peaks at the seafloor and decreases exponentially with height above bathymetry to a minimum of 5×10^{-6} m^2/s, with an e-folding scale of 150 m. The maximum values of the 3D vertical diffusivity ($\kappa^{3D}{}_{max}$) are 10^{-4}, 10^{-3}, and 3×10^{-3} m^2/s, respectively. According to previous studies (*18*, *57*–*59*), $\kappa^{3D}{}_{max} = 3 \times 10^{-3}$ m^2/s is much larger than observed, but $\kappa^{3D}{}_{max}$ in the range of 10^{-4} to 5×10^{-4} m^2/s is typical for the West Antarctic continental shelf and slope, and $\kappa^{3D}{}_{max} \sim 10^{-3}$ m^2/s has been observed over a ridge [figure S5 in (*57*)].

The barotropic tides in the Amundsen Sea are dominant by the diurnal tidal constituent K1 with a period of around 1 day and an amplitude of around 0.02 m/s in the deep ocean (*17*). In two perturbation simulations, idealized barotropic tidal currents are prescribed at the northern boundary, with a tidal period of 24 hours. The tidal current amplitudes prescribed at the northern boundary

are 0.025 and 0.05 m/s, respectively (table S1). Because of mass conservation, the resulting tidal current amplitudes are approximately 0.2 and 0.4 m/s over the shelf break in the two simulations, respectively (*60*).

Each simulation has been integrated for more than 12 model years, with a typical spin-up time of less than 7 years determined by the time series of domain-averaged kinetic energy, temperature, and salinity. The time average of the last 5 years is used for analysis.

Ice shelf melt rate sensitivity

Figure 3A shows that the change in bedrock elevation of the continental shelf (H_{bed}) exerts a relatively strong control on the undercurrent and ice shelf melt rate, because a larger H_{bed} corresponds to a thicker CDW layer over the shelf and a larger heat content. Through pumping warm water onto the shelf, tides of the typical amplitude observed in the Amundsen Sea (0.025 m/s in the deep ocean) increase ice shelf melt by 3.5 m/year (22%), consistent with previous estimates (*18*).

Below, we discuss the potential influence of zonal boundary conditions on the insensitivity of ice shelf melt rate to wind perturbation and the presence of a trough in the idealized model. An unexpected result (in light of our key findings) is that the ice shelf melt rate does not decrease when the trough is removed, although in this case, the eastward undercurrent transport is approximately 1/7 of the reference value (fig. S4C) and there is no southward CDW transport through the trough (Fig. 3F). This occurs because the coastal boundary current east of the ice shelf also transports heat toward the ice front (fig. S5, A and C), especially in the case without a trough. We find that there is a compensation between heat transport through the trough and by the coastal boundary current (fig. S5, B and D). As a result of this compensation, adding or removing a trough does not affect the melt rate. Since submarine troughs appear in the front of almost all major ice shelves, the simulations with a trough may be more relevant to nature. Furthermore, since multiple ice shelves exist in the Amundsen Sea, the coastal boundary current east of one ice shelf may have been transformed into a colder and fresher flow by upstream ice shelves. Nevertheless, this finding emphasizes the potentially important role of coastal boundary currents in ice shelf melt that needs to be understood thoroughly in the future.

The ice shelf melt rate is insensitive to variations in local wind speed, which might also be an artifact resulting from the zonal boundary conditions: Since the thermocline depth is relaxed toward a fixed curve at the zonal boundaries (Fig. 1D), the thickness of the CDW layer over the shelf is primarily influenced by boundary conditions rather than wind variations (*32*), which is potentially a caveat of this model. Figure S5 (G and H) indicates that with stronger surface northwestward winds, the trough heat transport decreases while the boundary current heat transport increases, leading to the same total CDW heat transport at the ice front and thus the same ice shelf melt rates.

Pseudo-ice shelf

To investigate the feedback between meltwater discharge and onshore heat transport carried by the slope undercurrent, we conducted simulations with a pseudo-ice shelf to control the melt rate explicitly. We add fixed temperature and salinity fluxes (sink of heat and dilution of salt) associated with ice shelf melt to the ocean thermodynamic equations. This is implemented by restoring temperature and salinity at the ice shelf-ocean interface, described below.

At the tilted interface between the ice shelf and the ocean, the temperature and salinity tendencies are modified so that

$$\frac{d\chi}{dt} \Rightarrow \frac{d\chi}{dt} - \frac{\chi - \chi_{relax}}{\tau_\chi} \tag{4}$$

where χ represents the potential temperature (T) or salinity (S) of the ocean, χ_{relax} is the relaxation value, and τ_χ is the relaxation timescale. We use an extremely long timescale $\tau_\chi = \tau_{inf} = 10^{20}$ s to relax temperature and salinity toward $\chi_{relax} = \tau_{inf}\, Q^{\chi}_{melt}$, where $Q^{\chi}_{melt} < 0$ represents the temperature and salinity fluxes due to ice shelf melt. This ensures that $|\chi| \ll |\chi_{relax}|$, and thus, the tendency terms become

$$\frac{d\chi}{dt} - \frac{\chi - \tau_{inf} Q^{\chi}_{melt}}{\tau_{inf}} \approx \frac{d\chi}{dt} + Q^{\chi}_{melt} \tag{5}$$

In the rest of this section, we estimate Q^{T}_{melt} and Q^{S}_{melt} by assuming a constant and uniform melt rate. As the ice shelf melts, the latent heat of fusion decreases ocean temperature beneath the ice shelf.

$$\rho_o c_p Q^{T}_{melt} \Delta z \approx -\omega_{melt} L_f \rho_o \tag{6}$$

where $c_p = 3974$ J/kg per °C is the specific heat capacity of seawater, $\Delta z = \Delta z(x, y) \approx 25$ to 29 m is the thickness of the grid cells right beneath the ice shelf, ω_{melt} is the prescribed basal melt rate in the unit of meter per second, and $L_f = 3.34 \times 10^5$ J/kg is the heat of fusion for ice. Therefore

$$Q^{T}_{melt}(x, y) = -\frac{\omega_{melt} L_f}{c_p\ \Delta z(x, y)} \tag{7a}$$

$$T_{relax} = \tau_{inf} Q^{T}_{melt} \tag{7b}$$

To a good approximation, the salinity of the ice shelf is zero (*49*). We assume that the mass of salt in the ocean layer right beneath the ice shelf (M_{salt}) is conserved during ice shelf melt, $M_{salt} = M_o S_{ref}$, where $M_o = \rho_o \Delta z \Delta A$ is the total ocean mass of that layer, ΔA is the horizontal area, and $S_{ref} = 34.3$ psu is the reference salinity in the unit of psu (practical salinity units). After an infinitesimal time step δt, the ice shelf meltwater has diluted ocean salinity by increasing the total mass of liquid from M_o to M_o'

$$M_o' = \rho_o \Delta z \Delta A + \rho_{melt} \omega_{melt} \Delta A \delta t \tag{8}$$

where $\rho_{melt} = 1000$ kg/m^3 is the reference density of meltwater. The salinity of these grid cells becomes $S' = M_{salt}/M_o'$. Given $\delta t \to 0$

$$Q^{S}_{melt}(x, y) = \frac{S' - S_{ref}}{\delta t} = -\frac{S_{ref}}{\delta t + \frac{\rho_o}{\rho_{melt}} \frac{\Delta z}{\omega_{melt}}} \approx -\frac{\omega_{melt} S_{ref} \rho_{melt}}{\rho_o \Delta z} \tag{9a}$$

$$S_{relax} = \tau_{inf} Q^{S}_{melt} \tag{9b}$$

We conducted simulations with prescribed melt rates of 0, 8, 16, and 24 m/year, respectively (see table S1). In all cases, the ice shelf thermodynamics were turned off. For the simulation with zero melt, no relaxation temperature nor salinity was prescribed at the tilted interface.

Depth-integrated CDW vorticity budget

Over the continental shelf and upper slope ($y < 270$ km and ocean depth < 3 km), the CDW layer (defined as water warmer than 0°C) directly sits over the topography. We vertically integrate the horizontal momentum equation from the seafloor [$z = \eta_b\ (x, y)$] to the upper bound of the CDW layer [$z = \eta_{iso}\ (x, y)$], the elevation of the isotherm between the CDW layer and the surface water)

$$\underbrace{\rho_0 \int_{\eta_b}^{\eta_{iso}} \frac{\partial \mathbf{u}}{\partial t} dz}_{\text{Tendency}} = \underbrace{-\int_{\eta_b}^{\eta_{iso}} \nabla_h p dz}_{\text{Pressure gradient force}} + \underbrace{\underbrace{\boldsymbol{\tau}_b}_{\text{Bottom friction}} + \underbrace{\mathcal{V}}_{\text{Viscous diffusion}}}_{\text{Dissipation}} + \underbrace{\rho_0 \int_{\eta_b}^{\eta_{iso}} \left(\underbrace{-f\hat{\mathbf{k}}\mathbf{u}}_{\text{Coriolis}} \underbrace{-\hat{\mathbf{k}}\zeta\mathbf{u}}_{\text{Vorticity adv.}} \underbrace{-w\partial_z \mathbf{u}}_{\text{Vertical adv.}} \underbrace{-\nabla_h \mathbf{u}^2/2}_{\text{KE gradient}} \right) dz}_{\text{Total advection}} \tag{10}$$

where $\mathbf{u} \equiv (u, v)$ is the horizontal velocity, w is the vertical velocity, $\nabla_h \equiv (\partial_x, \partial_y)$ is the horizontal gradient operator, $\hat{\mathbf{k}}$ is the unit vector along the z axis, and $\zeta = \hat{\mathbf{k}} \cdot (\nabla_h \times \mathbf{u})$ is the relative vorticity. The term $-(\mathbf{u}\cdot\nabla)\mathbf{u}$ is written as the sum of vorticity advection, vertical advection, and kinetic energy (KE) gradient. The Coriolis term is included in the total advection to be consistent with MITgcm's diagnostics.

To obtain a relatively smooth result of the CDW vorticity budget, we vertically interpolate every term in the momentum budget (Eq. 10) to a much finer vertical grid with ~3400 layers. Then, we take the curl of the steady-state ($\partial_t \mathbf{u} \equiv 0$) CDW momentum equation and apply the Leibniz integral rule to the pressure torque.

$$0 \approx \underbrace{J(p, \eta_{iso})|_{z=\eta_{iso}}}_{\text{Interfacial pres. torque}} \underbrace{-J(p, \eta_b)|_{z=\eta_b}}_{\text{Bottom pres. torque}} + \underbrace{\hat{\mathbf{k}}[\nabla_h(\boldsymbol{\tau}_b + \mathcal{V})]}_{\text{Dissipation}} + \underbrace{\hat{\mathbf{k}}(\nabla_h \rho_0 \int_{\eta_b}^{\eta_{iso}} \left[-f\hat{\mathbf{k}}\mathbf{u} - \hat{\mathbf{k}}\zeta\mathbf{u} - w\partial_z\mathbf{u} - \nabla_h \mathbf{u}^2/2)dz\right]}_{\text{Total advection}} \tag{11}$$

where $J(A, B) = (\partial_x A \partial_y B - \partial_x B \partial_y A)$ is the Jacobian operator and the equation $J(p|_{z=\eta}, \eta) = J(p, \eta)|_{z=\eta}$ has been applied to the derivation above ($\eta = \eta_b$ or η_{iso}). The total pressure torque is expressed as the sum of the BPT and the IPT between the CDW layer and the surface layer. In the open ocean where $y \geq 270$ km and ocean depth ≥ 3 km, the bottom layer with potential temperature lower than 0°C also exerts pressure torque on the CDW layer above. This bottom layer has negligible impact on the undercurrent and therefore is excluded from the analysis throughout this manuscript.

Pressure torques and vertical stretching of the CDW layer

The total pressure torque of the CDW layer is contributed by the BPT, IPT, and ice shelf pressure torque (Fig. 5). The ice shelf pressure torque only exists in a small portion of the inner ice shelf cavity, where the CDW layer is directly connected to the ice shelf (Fig. 5F). Most of the ice shelf cavity has a thin layer of meltwater at ocean surface that is colder than 0°C, which exerts an IPT to the CDW layer and balances the BPT.

Under the assumption of geostrophic balance, the BPT at the seafloor and IPT at the upper bound of the CDW layer are approximately

$$\text{BPT} = -J(p, \eta_b)|_{z=\eta_b} = -\rho_0 f(\mathbf{u}_g \cdot \nabla_h \eta_b)|_{z=\eta_b} \approx -\rho_0 f w_b \tag{12a}$$

$$\text{IPT} = J(p, \eta_{iso})|_{z=\eta_{iso}} = \rho_0 f(\mathbf{u}_g \cdot \nabla_h \eta_{iso})|_{z=\eta_{iso}} \approx \rho_0 f(w_{iso} - \omega^{dia}) \tag{12b}$$

where f is the Coriolis parameter, w is the vertical velocity, $\mathbf{u}_g = (u_g, v_g)$ is the geostrophic velocity, the subscripts $\bullet_b$ and $\bullet_{iso}$ denote seafloor and the interface between the CDW and surface water, respectively, and ω^{dia} is the diapycnal velocity across the 0°C isotherm. Therefore, the total pressure torque is approximately

$$\text{Total pressure torque} = \text{BPT} + \text{IPT} \approx \rho_0 f \left(\underbrace{w_{iso} - w_b}_{\text{Vertical stretching}} - \omega^{dia} \right) \tag{13}$$

The negative total pressure torque in the trough is generated by the vertical stretching of the CDW layer (Fig. 5A and fig. S3C).

In addition, as suggested by Stewart *et al.* (*37*), the total pressure torque can be written in the form of a horizontal transport term

$$\text{Total pressure torque} = J(p, \eta_{iso})|_{z=\eta_{iso}} - J(p, \eta_b)|_{z=\eta_b} = \nabla_h \cdot \underbrace{\left[(p\nabla_h^{\dagger}\eta_{iso})|_{z=\eta_{iso}} - (p\nabla_h^{\dagger}\eta_b)|_{z=\eta_b} \right]}_{\text{Pressure–torque vorticity flux}} \tag{14}$$

where $\nabla_h^{\dagger} = (\partial_y, -\partial_x)$ is the conjugate of ∇_h. The total pressure torque is the horizontal divergence of the pressure-torque vorticity flux.

For simulations with no trough, the undercurrent transport (almost zero) is not correlated with ice shelf melt (green and blue hollow markers in Fig. 3E), which is consistent with the transport of pressure-torque vorticity flux through the trough playing a key role in slope undercurrent generation. Furthermore, the simulations with a deeper thermocline (i.e., a steeper slope front) prescribed at the zonal boundaries do not fit closely with the theory described above. This is likely because the relatively strong westward flow in the upper layer arising from the steeper slope front prohibits the transfer of vorticity flux from the continental shelf to the continental slope. The undercurrent velocity in the simulation with a deeper thermocline and weaker winds (DeepWind_2) is relatively strong (Fig. 3E) because, in this case, the westward surface current is faster than the sea ice, resulting in an eastward momentum input to the ocean from the ice-ocean stress.

Coriolis term and diapycnal upwelling

The Coriolis term in the vorticity equation can be expressed as

$$\text{Coriolis} = -\hat{\mathbf{k}}\cdot(\nabla_h\times\rho_0\int_{\eta_b}^{\eta_{\text{iso}}} f\hat{\mathbf{k}}\times\mathbf{u}\,dz) = f\rho_0(\mathbf{u}\cdot\nabla_h\eta_b)|_{z=\eta_b} - f\rho_0(\mathbf{u}\cdot\nabla_h\eta_{\text{iso}})|_{z=\eta_{\text{iso}}} + f\rho_0(w_{\text{iso}} - w_b) - \rho_0\int_{\eta_b}^{\eta_{\text{iso}}}\beta v dz \approx f\rho_0\omega^{\text{dia}} - \rho_0\int_{\eta_b}^{\eta_{\text{iso}}}\beta v dz \quad (15)$$

where ω^{dia} is the diapycnal velocity across the upper bound of the CDW layer and $\beta = \partial_y f$ is the Rossby parameter. In the ice shelf cavity, the Coriolis term approximately equals $f\rho_0\omega^{\text{dia}}$, which is contributed by meltwater upwelling.

Note that in the case of a pseudo-ice shelf that does not melt, the weak undercurrent is associated with a standing wave that is anticyclonic to the north of the shelf break (Fig. 4A). This may be driven by the positive relative vorticity input from Coriolis advection associated with diapycnal downwelling at the mouth of the submarine trough near the shelf break (fig. S6G and Eq. 15).

Transport-weighted undercurrent velocity and cross-slope buoyancy gradient

The undercurrent transport in fig. S4C is defined as the zonal-average eastward transport over the shelf break west of the trough (-280 km $\leq x \leq 0$, 210 km $\leq y \leq 235$ km, $\eta_b \leq z \leq 0$)

$$\mathcal{T}^{\text{east}} = \frac{\iiint_{u>0} udV}{L_{x0}} \quad (16)$$

where L_{x0} = 280 km. The undercurrent strength is additionally quantified by the transport-weighted eastward velocity over the shelf break west of the trough (-280 km $\leq x \leq 0$, 210 km $\leq y \leq$ 235 km, $\eta_b \leq z \leq 0$, excluding the 20-km sponge layer at the western boundary)

$$\mathcal{U}^{\text{east}} = \frac{\iiint_{u>0} u^n \cdot udV}{\iiint_{u>0} u^n dV} \quad (n = 1) \quad (17)$$

In the two equations above, the latitudinal and longitudinal spans of the region are selected on the basis of the location of the undercurrent (fig. S2, C and F). We calculate the transport-weighted velocity (n = 1) instead of the traditionally defined volume-averaged velocity (n = 0) because, beyond the core of the undercurrent, a large portion of the slope has near zero eastward velocity (fig. S2, C, F, and I), leading to very small volume-averaged eastward velocities for all the simulations. Since the transport-weighted and volume-averaged velocities are highly correlated (r = 0.99), using the transport-weighted method does not affect the interpretation of the results but yields a better visual correspondence with cross sections of the undercurrent zonal velocity.

To quantify the cross-slope buoyancy gradient, we first calculate the geostrophic velocity by vertically integrating the thermal wind shear over the shelf break, with the assumption of zero bottom velocity (Eq. 18a), and then calculate the volume average (Eq. 18b, referred to as "thermal wind velocity" in the manuscript). We use the volume average instead of the transport-weighted method in Eq. 18b to estimate the bulk buoyancy gradient.

$$u_g(x, y, z) = \int_{\eta_b}^{z} \frac{g}{\rho_0 f}\frac{\partial\rho}{\partial y}dz' \quad (18a)$$

$$\mathcal{U}_g^{\text{east}} = \frac{\iiint_{u_g>0,\ \text{shelf break}} u_g dV}{\iiint_{u_g>0,\ \text{shelf break}} dV} \quad (18b)$$

Figure S4 shows that stronger ice shelf melt leads to a larger cross-slope buoyancy gradient (fig. S4B) and drives an undercurrent that approximately follows the thermal wind relation (fig. S4E). Note that the thermal wind velocity is about five times weaker than the undercurrent velocity (fig. S4E), and therefore, the undercurrent dynamics could not be simply explained by the baroclinic structure of the slope front. Stronger undercurrent velocities are associated with stronger shelf-break pressure torques, indicating the topographic control of the undercurrent strength (fig. S4D).

Shoreward CDW transport within the trough

The shoreward CDW transport within the trough (Fig. 3F) is calculated by integrating the southward velocity ($v < 0$) across -50 km $\leq x \leq$ 50 km, 100 km $\leq y \leq$ 225 km, and $\eta_b \leq z \leq \eta_{\text{cdw}}$ and then divided by the length of the trough (L_{trough} = 125 km)

$$\mathcal{T}_{\text{trough}}^{\text{south}} = \frac{\iiint_{v<0} vdV}{L_{\text{trough}}} \quad (19)$$

Supplementary Materials

This PDF file includes:
Figs. S1 to S6
Table S1
References

REFERENCES AND NOTES

1. F. S. Paolo, H. A. Fricker, L. Padman, Volume loss from Antarctic ice shelves is accelerating. *Science* **348**, 327–331 (2015).
2. Y. Liu, J. C. Moore, X. Cheng, R. M. Gladstone, J. N. Bassis, H. Liu, J. Wen, F. Hui, Ocean-driven thinning enhances iceberg calving and retreat of Antarctic ice shelves. *Proc. Natl. Acad. Sci. U.S.A.* **112**, 3263–3268 (2015).
3. E. Rignot, S. Jacobs, J. Mouginot, B. Scheuchl, Ice-shelf melting around Antarctica. *Science* **341**, 266–270 (2013).
4. H. Pritchard, S. R. Ligtenberg, H. A. Fricker, D. G. Vaughan, M. R. van den Broeke, L. Padman, Antarctic ice-sheet loss driven by basal melting of ice shelves. *Nature* **484**, 502–505 (2012).
5. S. S. Jacobs, A. Jenkins, C. F. Giulivi, P. Dutrieux, Stronger ocean circulation and increased melting under Pine Island Glacier ice shelf. *Nat. Geosci.* **4**, 519–523 (2011).
6. G. H. Gudmundsson, F. S. Paolo, S. Adusumilli, H. A. Fricker, Instantaneous Antarctic ice sheet mass loss driven by thinning ice shelves. *Geophys. Res. Lett.* **46**, 13903–13909 (2019).
7. R. M. DeConto, Coauthors., The Paris climate agreement and future sea-level rise from Antarctica. *Nature* **593**, 83–89 (2021).
8. I. Joughin, D. Shapero, P. Dutrieux, B. Smith, Ocean-induced melt volume directly paces ice loss from Pine Island Glacier. *Sci. Adv.* **7**, eabi5738 (2021).
9. I. Joughin, R. B. Alley, Stability of the West Antarctic ice sheet in a warming world. *Nat. Geosci.* **4**, 506–513 (2011).
10. D. P. Walker, A. Jenkins, K. M. Assmann, D. R. Shoosmith, M. A. Brandon, Oceanographic observations at the shelf break of the Amundsen Sea, Antarctica. *J. Geophys. Res. Oceans* **118**, 2906–2918 (2013).
11. J. E. Hazel, A. L. Stewart, Are the near-Antarctic easterly winds weakening in response to enhancement of the Southern Annular Mode? *J. Climate* **32**, 1895–1918 (2019).

12. I. Robinson, Tidal vorticity and residual circulation. *Deep Sea Res. I Oceanogr. Res. Pap.* **28**, 195–212 (1981).
13. A. Silvano, P. R. Holland, K. A. Naughten, O. Dragomir, P. Dutrieux, A. Jenkins, Y. Si, A. L. Stewart, B. P. Molino, G. W. Janzing, T. S. Dotto, A. C. Naveira Garabato, Baroclinic ocean response to climate forcing regulates decadal variability of ice-shelf melting in the Amundsen Sea. *Geophys. Res. Lett.* **49**, e2022GL100646 (2022).
14. S. Kimura, A. Jenkins, H. Regan, P. R. Holland, K. M. Assmann, D. B. Whitt, M. Van Wessem, W. J. van de Berg, C. H. Reijmer, P. Dutrieux, Oceanographic controls on the variability of ice-shelf basal melting and circulation of glacial meltwater in the Amundsen Sea Embayment, Antarctica. *J. Geophys. Res. Oceans* **122**, 10131–10155 (2017).
15. M. Azaneu, B. Webber, K. J. Heywood, K. M. Assmann, T. S. Dotto, E. P. Abrahamsen, Influence of shelf break processes on the transport of warm waters onto the eastern Amundsen Sea continental shelf. *J. Geophys. Res. Oceans* **128**, e2022JC019535 (2023).
16. K. A. Naughten, P. R. Holland, P. Dutrieux, S. Kimura, D. T. Bett, A. Jenkins, Simulated twentieth-century ocean warming in the Amundsen Sea, West Antarctica. *Geophys. Res. Lett.* **49**, e2021GL094566 (2022).
17. N. C. Jourdain, J.-M. Molines, J. Le Sommer, P. Mathiot, J. Chanut, C. de Lavergne, G. Madec, Simulating or prescribing the influence of tides on the Amundsen Sea ice shelves. *Ocean Model.* **133**, 44–55 (2019).
18. R. Robertson, Tidally induced increases in melting of Amundsen Sea ice shelves. *J. Geophys. Res. Oceans* **118**, 3138–3145 (2013).
19. T. S. Dotto, A. C. N. Garabato, A. K. Wåhlin, S. Bacon, P. R. Holland, S. Kimura, M. Tsamados, L. Herraiz-Borreguero, O. Kalén, A. Jenkins, Control of the oceanic heat content of the Getz-Dotson Trough, Antarctica, by the Amundsen Sea Low. *J. Geophys. Res. Oceans* **125**, e2020JC016113 (2020).
20. T. S. Dotto, A. C. N. Garabato, S. Bacon, P. R. Holland, S. Kimura, Y. L. Firing, M. Tsamados, A. K. Wåhlin, A. Jenkins, Wind-driven processes controlling oceanic heat delivery to the Amundsen Sea, Antarctica. *J. Phys. Oceanogr.* **49**, 2829–2849 (2019).
21. P. R. Holland, T. J. Bracegirdle, P. Dutrieux, A. Jenkins, E. J. Steig, West Antarctic ice loss influenced by internal climate variability and anthropogenic forcing. *Nat. Geosci.* **12**, 718–724 (2019).
22. M. Donat-Magnin, N. C. Jourdain, P. Spence, J. Le Sommer, H. Gall'ee, G. Durand, Ice-shelf melt response to changing winds and glacier dynamics in the Amundsen Sea sector, Antarctica. *J. Geophys. Res. Oceans* **122**, 10206–10224 (2017).
23. C. M. Little, A. Gnanadesikan, M. Oppenheimer, How ice shelf morphology controls basal melting. *J. Geophys. Res. Oceans* **114**, C12007 (2009).
24. P. Dutrieux, J. De Rydt, A. Jenkins, P. R. Holland, H. K. Ha, S. H. Lee, E. J. Steig, Q. Ding, E. P. Abrahamsen, M. Schröder, Strong sensitivity of Pine Island ice-shelf melting to climatic variability. *Science* **343**, 174–178 (2014).
25. P. Mathiot, A. Jenkins, C. Harris, G. Madec, Explicit representation and parametrised impacts of under ice shelf seas in the z^* coordinate ocean model NEMO 3.6. *Geosci. Model Dev.* **10**, 2849–2874 (2017).
26. A. T. Bradley, D. T. Bett, P. Dutrieux, J. De Rydt, P. R. Holland, The influence of Pine Island Ice Shelf calving on basal melting. *J. Geophys. Res. Oceans* **127**, e2022JC018621 (2022).
27. N. C. Jourdain, P. Mathiot, N. Merino, G. Durand, J. Le Sommer, P. Spence, P. Dutrieux, G. Madec, Ocean circulation and sea-ice thinning induced by melting ice shelves in the Amundsen Sea. *J. Geophys. Res. Oceans* **122**, 2550–2573 (2017).
28. R. Chen, J. C. McWilliams, L. Renault, Momentum Governors of California Undercurrent Transport. *J. Phys. Oceanogr.* **51**, 2915–2932 (2021).
29. J. Li, J. Gan, On the formation dynamics of the north equatorial undercurrent. *J. Phys. Oceanogr.* **50**, 1399–1415 (2020).
30. J. Schaffer, R. Timmermann, J. E. Arndt, S. S. Kristensen, C. Mayer, M. Morlighem, D. Steinhage, A global, high-resolution data set of ice sheet topography, cavity geometry, and ocean bathymetry. *Earth Syst. Sci. Data* **8**, 543–557 (2016).
31. A. F. Thompson, A. L. Stewart, P. Spence, K. J. Heywood, The Antarctic Slope Current in a changing climate. *Rev. Geophys.* **56**, 741–770 (2018).
32. A. Jenkins, P. Dutrieux, S. Jacobs, E. J. Steig, G. H. Gudmundsson, J. Smith, K. J. Heywood, Decadal ocean forcing and Antarctic ice sheet response: Lessons from the Amundsen Sea. *Oceanography* **29**, 106–117 (2016).
33. P. M. Hampson, J. M. Pringle, Glacial troughs enhance shelf/slope exchange in the barotropic limit. *J. Geophys. Res. Oceans* **127**, e2021JC018207 (2022).
34. S. Adusumilli, H. A. Fricker, B. Medley, L. Padman, M. R. Siegfried, Interannual variations in meltwater input to the Southern Ocean from Antarctic ice shelves. *Nat. Geosci.* **13**, 616–620 (2020).
35. A. F. Thompson, K. G. Speer, L. M. Schulze Chretien, Genesis of the Antarctic slope current in West Antarctica. *Geophys. Res. Lett.* **47**, e2020GL087802 (2020).
36. M. M. Flexas, A. F. Thompson, M. P. Schodlok, H. Zhang, K. Speer, Antarctic Peninsula warming triggers enhanced basal melt rates throughout West Antarctica. *Sci. Adv.* **8**, eabj9134 (2022).
37. A. L. Stewart, J. C. McWilliams, A. Solodoch, On the role of bottom pressure torques in wind-driven gyres. *J. Phys. Oceanogr.* **51**, 1441–1464 (2021).
38. D. T. Bett, P. R. Holland, A. C. Naveira Garabato, A. Jenkins, P. Dutrieux, S. Kimura, A. Fleming, The impact of the Amundsen Sea freshwater balance on ocean melting of the West Antarctic Ice Sheet. *J. Geophys. Res. Oceans* **125**, e2020JC016305 (2020).
39. A. K. Wåhlin, O. Kalén, K. M. Assmann, E. Darelius, H. K. Ha, T. W. Kim, S. H. Lee, Subinertial oscillations on the Amundsen Sea Shelf, Antarctica. *J. Phys. Oceanogr.* **46**, 2573–2582 (2016).
40. M. Haigh, P. R. Holland, A. Jenkins, The influence of bathymetry over heat transport onto the Amundsen Sea continental shelf. *J. Geophys. Res. Oceans* **128**, e2022JC019460 (2023).
41. P. St-Laurent, J. M. Klinck, M. S. Dinniman, On the role of coastal troughs in the circulation of warm circumpolar deep water on Antarctic shelves. *J. Phys. Oceanogr.* **43**, 51–64 (2013).
42. J. Marshall, A. Adcroft, C. Hill, L. Perelman, C. Heisey, A finite-volume, incompressible Navier Stokes model for studies of the ocean on parallel computers. *J. Geophys. Res.* **102**, 5753–5766 (1997).
43. J. Marshall, C. Hill, L. Perelman, A. Adcroft, Hydrostatic, quasi-hydrostatic, and nonhydrostatic ocean modeling. *J. Geophys. Res.* **102**, 5733–5752 (1997).
44. T. J. McDougall, D. R. Jackett, D. G. Wright, R. Feistel, Accurate and computationally efficient algorithms for potential temperature and density of seawater. *J. Atmos. Oceanic Tech.* **20**, 730–741 (2003).
45. W. D. Hibler III, A dynamic thermodynamic sea ice model. *J. Phys. Oceanogr.* **9**, 815–846 (1979).
46. W. D. Hibler III, Modeling a variable thickness sea ice cover. *Mon. Weather Rev.* **108**, 1943–1973 (1980).
47. M. Winton, A reformulated three-layer sea ice model. *J. Atmos. Oceanic Tech.* **17**, 525–531 (2000).
48. M. Losch, D. Menemenlis, J.-M. Campin, P. Heimbach, C. Hill, On the formulation of sea-ice models. Part 1: Effects of different solver implementations and parameterizations. *Ocean Model.* **33**, 129–144 (2010).
49. D. M. Holland, A. Jenkins, Modeling thermodynamic ice–ocean interactions at the base of an ice shelf. *J. Phys. Oceanogr.* **29**, 1787–1800 (1999).
50. D. E. Gwyther, K. Kusahara, X. S. Asay-Davis, M. S. Dinniman, B. K. Galton-Fenzi, Vertical processes and resolution impact ice shelf basal melting: A multi-model study. *Ocean Model.* **147**, 101569 (2020).
51. R. A. Locarnini, Coauthors. World Ocean Atlas 2023, Volume 1: Temperature (2023).
52. E. Randall-Goodwin, M. P. Meredith, A. Jenkins, P. L. Yager, R. M. Sherrell, E. P. Abrahamsen, R. Guerrero, X. Yuan, R. A. Mortlock, K. Gavahan, A.-C. Alderkamp, H. Ducklow, R. Robertson, S. E. Stammerjohn, Freshwater distributions and water mass structure in the Amundsen Sea Polynya region, Antarctica. *Elem. Sci. Anth.* **3**, 000065 (2015).
53. Y. Si, A. Stewart, I. Eisenman, Coupled ocean/sea ice dynamics of the Antarctic Slope Current driven by topographic eddy suppression and sea ice momentum redistribution. *J. Phys. Oceanogr.* **52**, 1563–1589 (2022).
54. J. Smagorinsky, General circulation experiments with the primitive equations. *Mon. Weather Rev.* **91**, 99–164 (1963).
55. J. Smagorinsky, Some historical remarks on the use of nonlinear viscosities, in *Evolution of Physical Oceanography*, B. Galperin, S. Orszag, Eds. (Cambridge Univ. Press, 1993), pp. 3–36.
56. S. M. Griffies, R. W. Hallberg, Biharmonic friction with a Smagorinsky-like viscosity for use in large-scale eddy-permitting ocean models. *Mon. Weather Rev.* **128**, 2935–2946 (2000).
57. R. M. Scott, J. A. Brearley, A. C. Naveira Garabato, H. J. Venables, M. P. Meredith, Rates and mechanisms of turbulent mixing in a coastal embayment of the West Antarctic Peninsula. *J. Geophys. Res. Oceans* **126**, e2020JC016861 (2021).
58. J. A. Brearley, M. P. Meredith, A. C. N. Garabato, H. J. Venables, M. E. Inall, Controls on turbulent mixing on the West Antarctic Peninsula shelf. *Deep Sea Res. II Top. Stud. Oceanogr.* **139**, 18–30 (2017).
59. S. Howard, J. Hyatt, L. Padman, Mixing in the pycnocline over the western Antarctic Peninsula shelf during Southern Ocean GLOBEC. *Deep Sea Res. II Top. Stud. Oceanogr.* **51**, 1965–1979 (2004).
60. J. W. Loder, Topographic rectification of tidal currents on the sides of Georges Bank. *J. Phys. Oceanogr.* **10**, 1399–1416 (1980).
61. J. Towns, T. Cockerill, M. Dahan, I. Foster, K. Gaither, A. Grimshaw, V. Hazlewood, S. Lathrop, D. Lifka, G. D. Peterson, R. Roskies, J. R. Scott, N. Wilkins-Diehr, XSEDE: Accelerating scientific discovery. *Comput. Sci. Eng.* **16**, 62–74 (2014).
62. T. W. K. Armitage, R. Kwok, A. F. Thompson, G. Cunningham, Dynamic topography and sea level anomalies of the Southern Ocean: Variability and teleconnections. *J. Geophys. Res. Oceans* **123**, 613–630 (2018).
63. S. A. Good, M. J. Martin, N. A. Rayner, En4: Quality controlled ocean temperature and salinity profiles and monthly objective analyses with uncertainty estimates. *J. Geophys. Res. Oceans* **118**, 6704–6716 (2013).
64. A. Jenkins, D. Shoosmith, P. Dutrieux, S. Jacobs, T. W. Kim, S. H. Lee, H. K. Ha, S. Stammerjohn, West Antarctic Ice Sheet retreat in the Amundsen Sea driven by decadal oceanic variability. *Nat. Geosci.* **11**, 733–738 (2018).

65. C. Amante, B. W. Eakins, ETOPO1, 1 Arc-Minute Global Relief Model. Procedures, Data Sources and Analysis. NOAA Technical Memorandum NESDIS NGDC-24. National Geophysical Data Center, NOAA (2009).

Acknowledgments: This work used the Hoffman2 Cluster, which is supported by the IDRE Research Technology Group at UCLA. This work also used the Extreme Science and Engineering Discovery Environment (XSEDE) (*61*), which is supported by the National Science Foundation grant number ACI-1548562. We thank the MITgcm team for contribution to numerical modeling and for making their code available. We thank the editor and reviewers for insightful comments and suggestions. **Funding:** This work was supported by National Science Foundation grant OCE-1751386 (A.L.S.), National Science Foundation grant OPP-2220968 (A.L.S.), National Science Foundation grant ACI -548562 (A.L.S.), and Natural Environment Research Council (NE/V014285/1) (A.S.). **Author contributions:** Conceptualization: A.L.S., Y.S., A.S., and A.C.N.G. Methodology: Y.S. and A.L.S. Investigation: Y.S. Visualization: Y.S. Supervision: A.S. and A.L.S. Writing—original draft: A.C.N.G. and Y.S. Writing—review and editing: A.L.S., A.S., and A.C.N.G. **Competing interests:** The authors declare that they have no competing interests. **Data and materials availability:** The source code of the MITgcm is available at https://mitgcm.org. A copy of the MITgcm source code, the MATLAB scripts used to generate, run, and analyze the MITgcm simulations, the products that we calculated from MITgcm model diagnostics, and the data we used to make figures for this manuscript are available at http://doi.org/10.5281/zenodo.8239814. All the raw data of the model output are available at https://doi.org/10.15144/S4QP4N. All data needed to evaluate the conclusions in the paper are present in the paper and/or the Supplementary Materials.

Submitted 10 October 2023
Accepted 15 March 2024
Published 17 April 2024
10.1126/sciadv.adl0601
