# Supplementary material for: Antarctic Slope Undercurrent and onshore heat transport driven by ice shelf melting
Source: Sci Adv. 2024 Apr 17;10(16):eadl0601. doi: 10.1126/sciadv.adl0601 (PMC11650804; doi:10.1126/sciadv.adl0601)
Supplement: Supplementary file 2 — Figs. S1 to S6 Table S1 References [file sciadv.adl0601_sm.pdf]

Science Advances
AAAS

Supplementary Materials for

Antarctic Slope Undercurrent and onshore heat transport driven by ice shelf melting

Yidongfang Si *et al.*

Corresponding author: Yidongfang Si, y_si@mit.edu

Sci. Adv. **10**, eadl0601 (2024)
DOI: 10.1126/sciadv.adl0601

This PDF file includes:

Figs. S1 to S6
Table S1
References

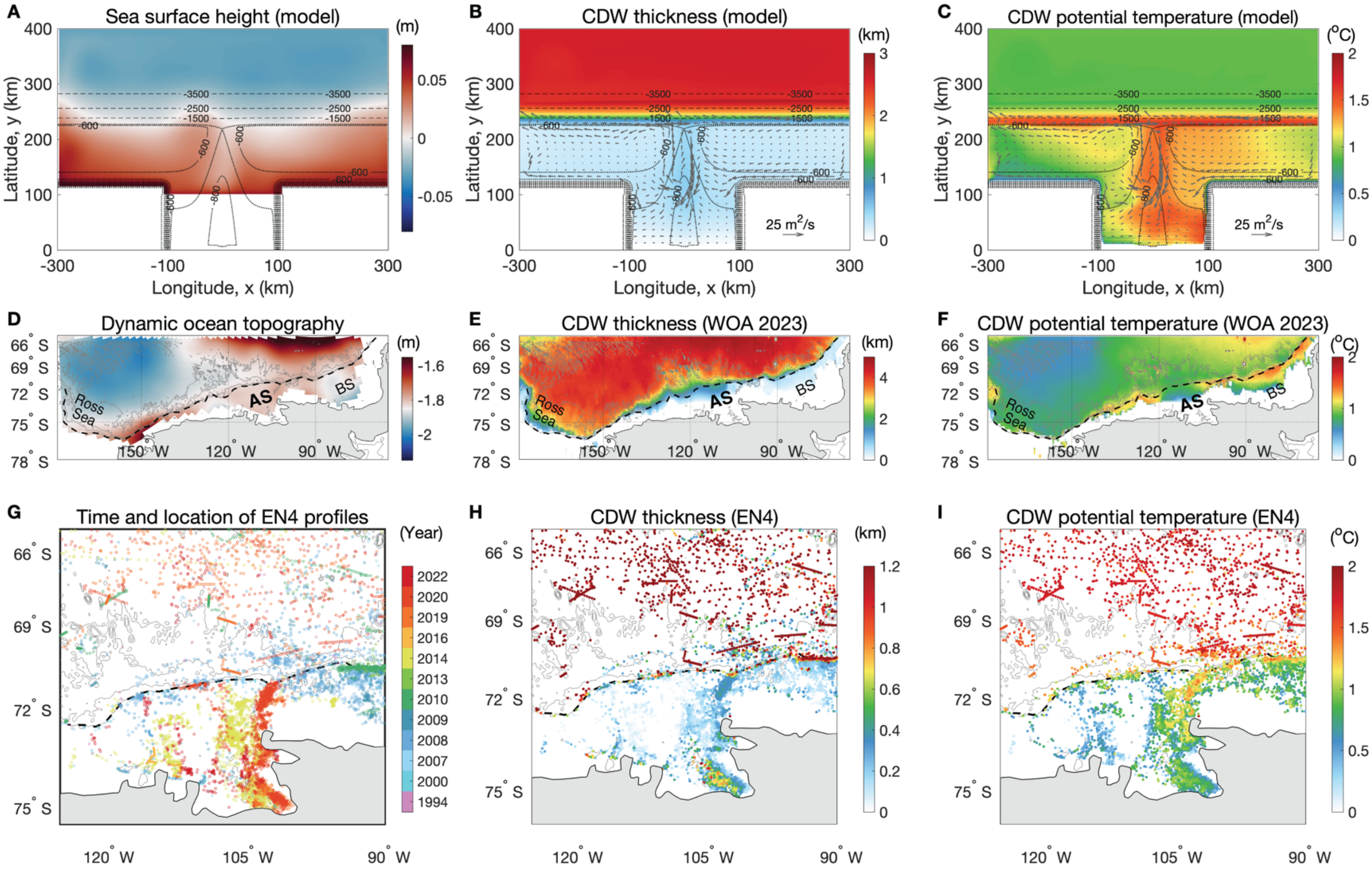

Fig. S1. Comparison of the reference simulation with observations. (**A**) Sea surface height anomaly, (**B**) thickness of the Circumpolar Deep Water (CDW) layer, and (**C**) depth-averaged CDW potential temperature in the reference simulation, overlaid by gray arrows of CDW volume flux over the shelf and upper slope ($y \leq 250$ km). The bathymetric contours are denoted by the thin dashed lines with an interval of 1000 m, and thin dotted lines with an interval of 100 m. (**D**) Mean dynamic ocean topography of the Ross Sea, the Amundsen Sea (AS), and the Bellingshausen Sea (BS) averaged from 2011 to 2013. Data taken from Armitage et al. (2018) (62). (**E**)-(**F**) Climatological (1991–2020) mean CDW thickness and mean CDW potential temperature from the World Ocean Atlas (WOA) 2023 (51). (**G**) Time and location of quality-controlled ocean temperature profiles over the Amundsen Sea continental shelf since 1994. The data come from EN4 dataset (EN.4.2.2) by Good et al. (2013) (63). Only the years/months with observations of CDW over the Amundsen Sea continental shelf are included: Feb. to Mar. of 1994, Jan. to Mar. of 2000, Feb. to Apr. and Dec. of 2007, Mar. to Jun. and Dec. of 2008, Jan. to Apr. of 2009, Mar. to Sept. of 2010, Dec. of 2013, Jan. to Oct. of 2014, Jan. to Feb. of 2016, Feb. to Nov. of 2019, Mar. to Sept. of 2020, and Feb. to Sept. of 2022. (**H**) CDW thickness in the EN4 dataset. Note that the colorbar of panel (H) is saturated to highlight the variation of CDW thickness over the continental shelf. (**I**) CDW potential temperature in the EN4 dataset. The modeled CDW temperature over the shelf is comparable to Jacobs et al. (2011) (5), but is warmer than the EN4 profiles. The discrepancy is probably due to the strong decadal oceanic variability (64) and the limited seasons for observations. In panels d–i, the thin gray contours denote the ocean bathymetry with an interval of 1000 m and the dashed black curve denotes the shelf break (ocean depth equals 1000 m). The bathymetric data is taken from Amante and Eakins (2009) (65).

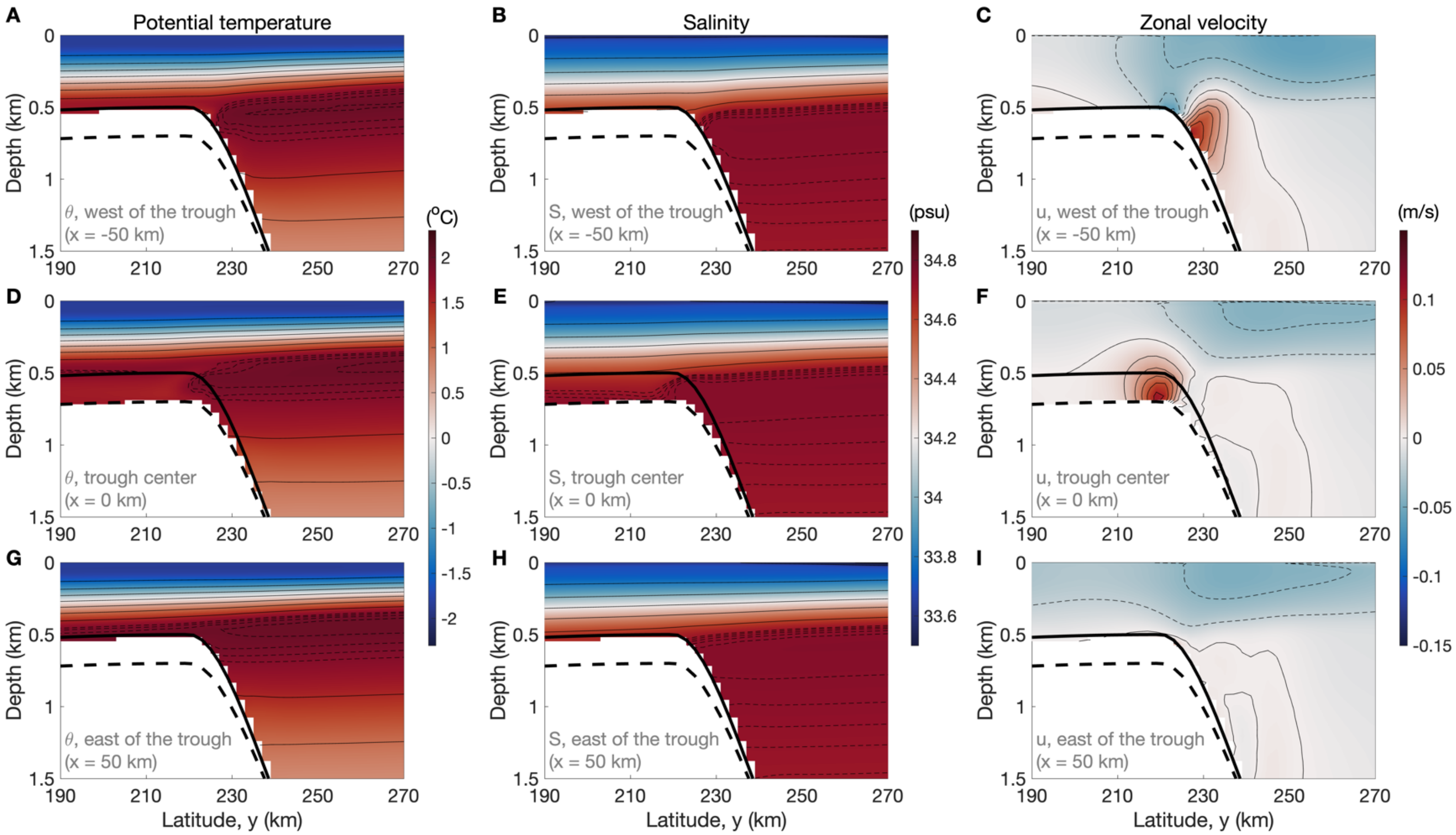

Fig. S2. Cross-sections of potential temperature, salinity, and zonal velocity. (**A**)-(**C**) West of the trough at $x = -50$ km. (**D**)-(**F**) Trough center at $x = 0$ km. (**G**)-(**I**) East of the trough at $x = 50$ km. The shallowest and deepest bathymetric contours are indicated by the thick solid and dashed lines, respectively.

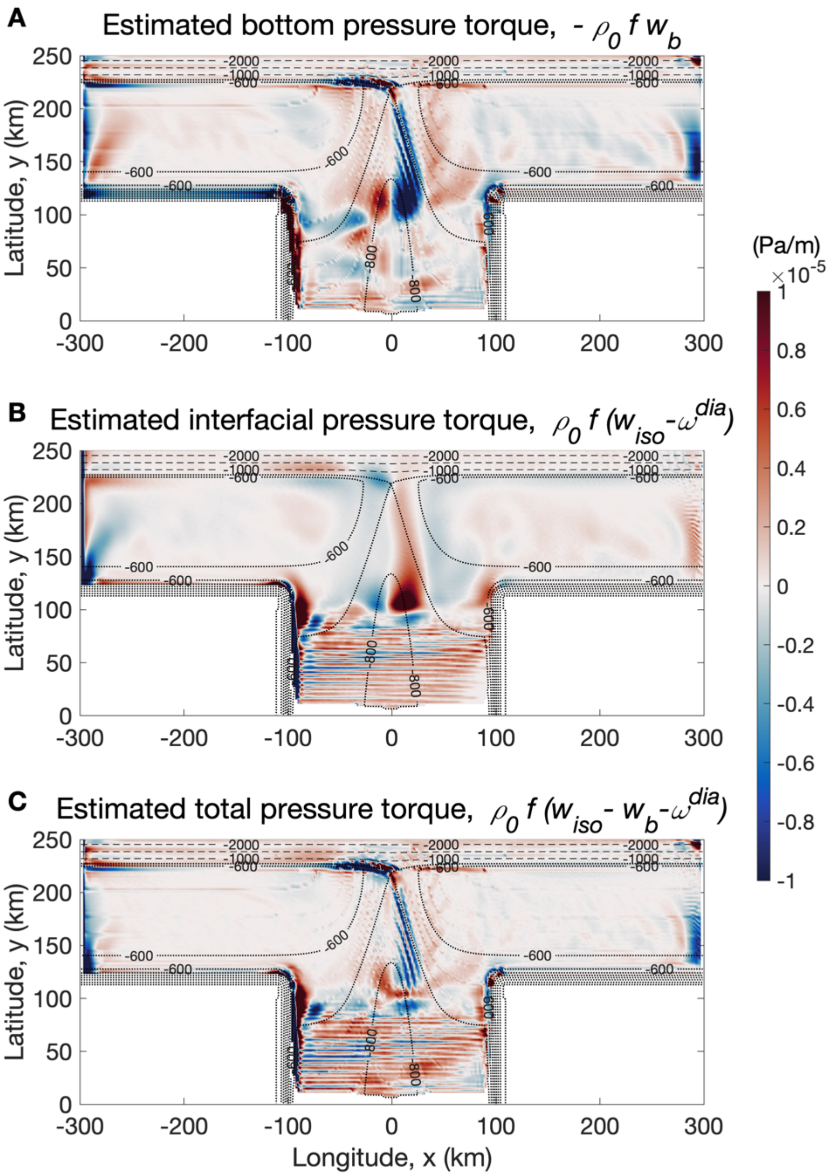

Fig. S3. Estimated pressure torques of the Circumpolar Deep Water (CDW) layer using Eqs. (12)–(13). (**A**) Estimated bottom pressure torque. (**B**) Estimated interfacial pressure torque at the upper bound of the CDW layer. (**C**) Estimated total pressure torque, which is the sum of panels (A) and (B).

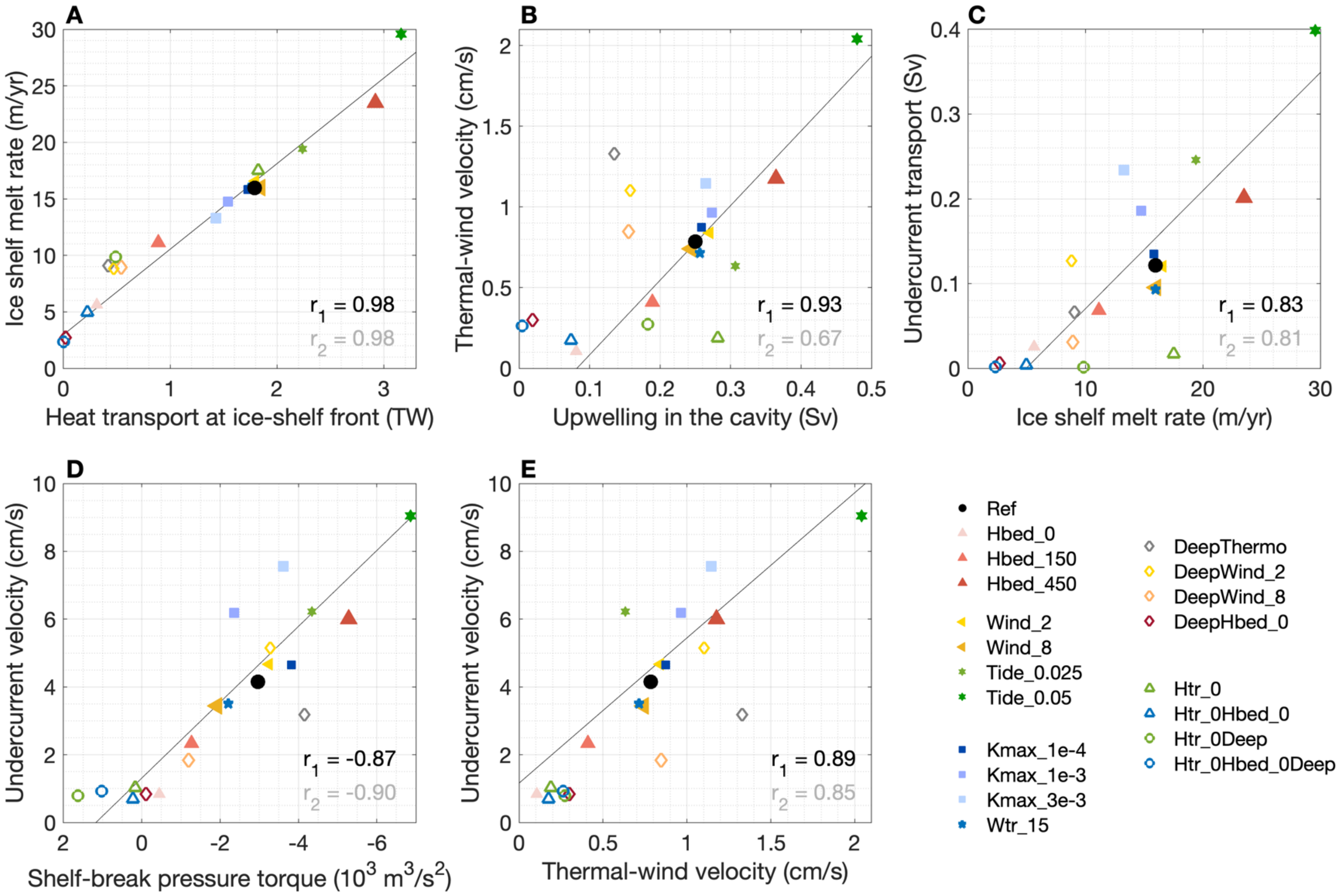

Fig. S4. Perturbation experiments. (**A**) Correlation between the cumulative heat transport at the ice shelf front (y = 100 km) with ice shelf melt rate. (**B**) Correlation between the diapycnal upwelling across the 0◦C isotherm in the ice-shelf cavity with the thermal-wind velocity, which is a quantification of the cross-slope buoyancy gradient (Eq. 18b). (**C**) Correlation between ice shelf melt rate and zonal-mean undercurrent transport west of the trough (1 Sv = 10^6 m/s; Eq. 16). (**D**)-(**E**) Correlation between the transport-weighted undercurrent velocity (Eq. 17) with the pressure torque of the Circumpolar Deep Water layer integrated over the shelf break west of the trough (210 km $\leqslant y \leqslant$ 235 km, -120 km $\leqslant x \leqslant$ 0 km, the green box in Fig. 5J) and thermal-wind velocity, respectively. In each panel, r_1 is the correlation coefficient for simulations with fixed topographic geometry and boundary isopycnal geometry, denoted by the filled markers; r_2 is the correlation coefficient for all simulations, including simulations with varied topographic geometry and boundary isopycnal geometry, denoted by the hollow markers; the thin black line is the linear fit for the filled markers. The names of the experiments in the figure are consistent with table S1.

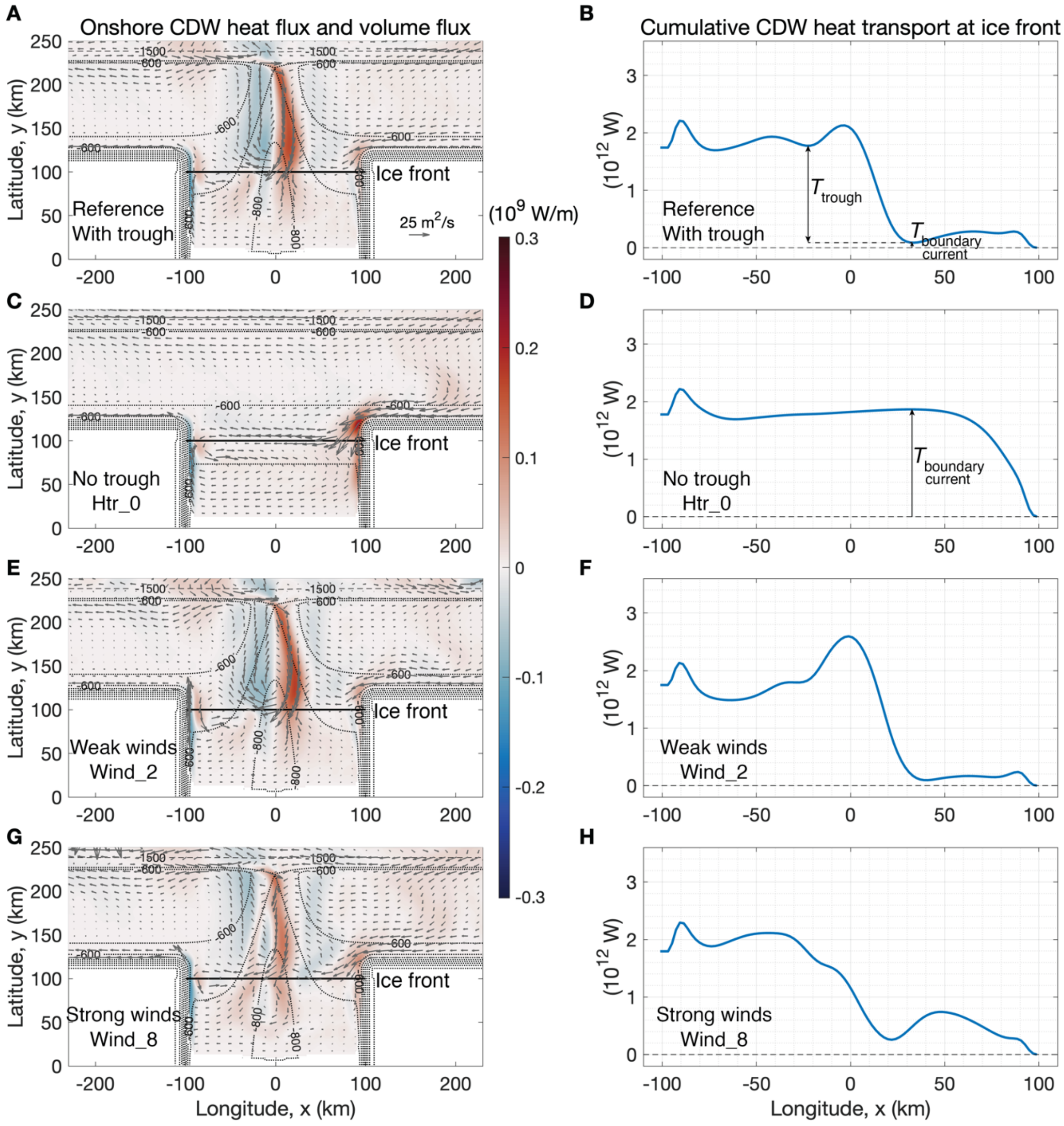

Fig. S5. A compensation between heat transport through the trough and by the boundary current. Left column: Vertically integrated heat flux (color) and volume flux (arrows) in the Circumpolar Deep Water (CDW) layer, with red corresponds to shoreward (southward) heat flux. The black solid line denotes the location of the ice shelf front at y = 100 km. The bathymetric contours are denoted by the thin dashed lines with an interval of 1000 m, and thin dotted lines with an interval of 100 m. Right column: Cumulative shoreward CDW heat transport at the ice shelf front as a function of longitude. The CDW heat flux is first integrated vertically and then integrated zonally along the ice shelf front, from x = 100 km to x = −100 km. (**A**)-(**B**) The reference simulation. T_{trough} is the heat carried by the undercurrent through the trough. $T_{\text{boundary current}}$ is the heat carried by the coastal boundary current. (**C**)-(**D**) The simulation with no trough. (**E**)-(**F**) The simulation with weak surface winds (U_{a0} = −2 m/s, V_{a0} = 2 m/s). (**G**)-(**H**) The simulation with strong surface winds (U_{a0} = −8 m/s, V_{a0} = 8 m/s).

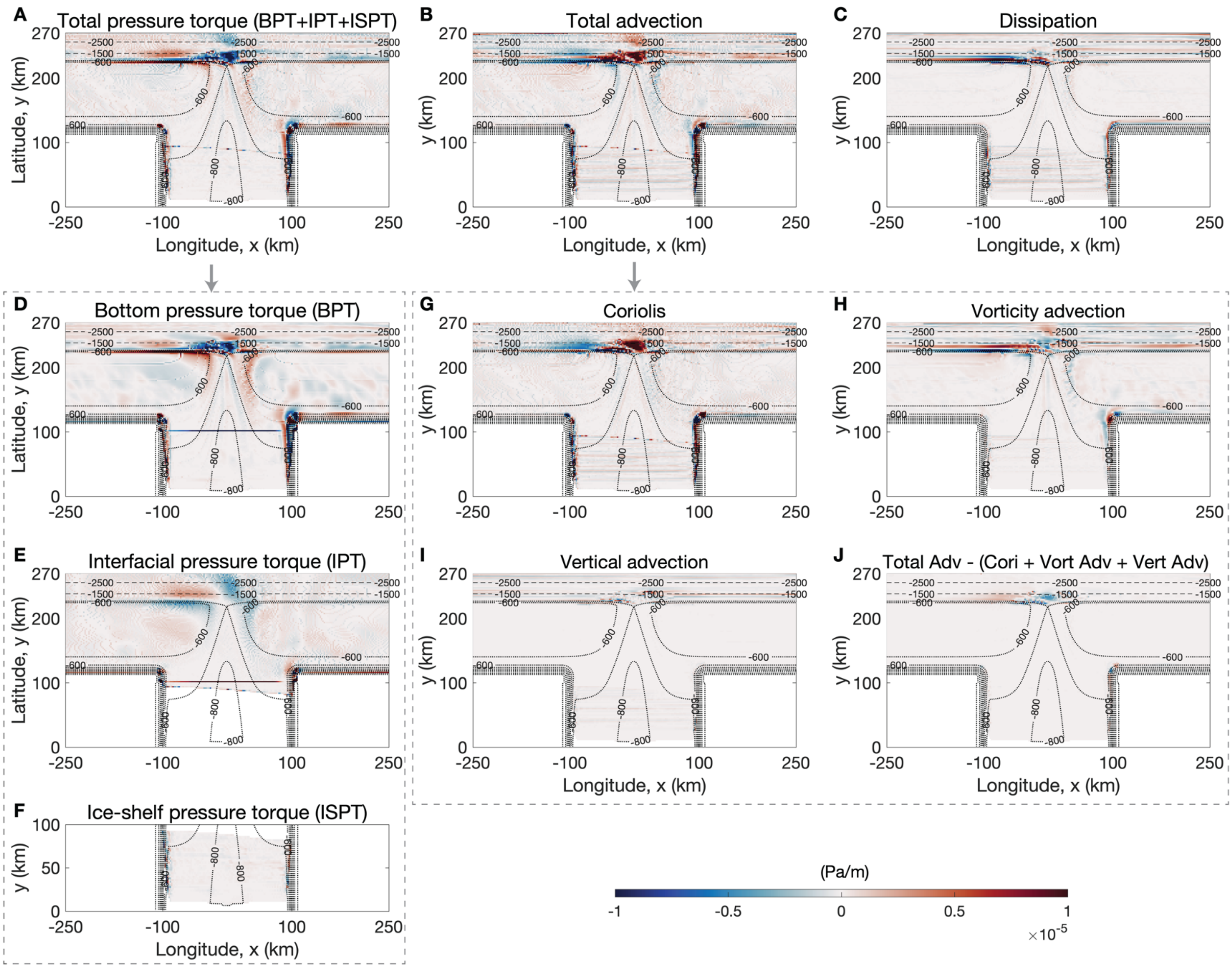

Fig. S6. Vorticity budget of the Circumpolar Deep Water layer for the pseudo ice shelf simulation with no melt. Figure labels are the same as Fig. 5.

Short name	h_{i0} (m)	(U_{a0}, V_{a0}) (m/s)	A_{tide} (m/s)	W_{tr} (km)	H_{bed} (m)	H_{tr} (m)	Z_{sb} (m)	Z_s (m)	κ^{3D}_{max} (m^2/s)	Ice shelf	ω_{melt} (m/s)
Reference	**1.0**	**(-5, 5)**	**0**	**30**	**300**	**200**	**550**	**700**	–	**Real**	–
Wind_2	1.0	**(-2, 2)**	0	30	300	200	550	700	–	Real	–
Wind_8	1.0	**(-8, 8)**	0	30	300	200	550	700	–	Real	–
Hbed_0	1.0	(-5, 5)	0	30	**0**	200	550	700	–	Real	–
Hbed_150	1.0	(-5, 5)	0	30	**150**	200	550	700	–	Real	–
Hbed_450	1.0	(-5, 5)	0	30	**450**	200	550	700	–	Real	–
Wtr_15	1.0	(-5, 5)	0	**15**	300	200	550	700	–	Real	–
Tide_0.025	1.0	(-5, 5)	**0.025**	30	300	200	550	700	–	Real	–
Tide_0.05	1.0	(-5, 5)	**0.05**	30	300	200	550	700	–	Real	–
Kmax_1e-4	1.0	(-5, 5)	0	30	300	200	550	700	**0.0001**	Real	–
Kmax_1e-3	1.0	(-5, 5)	0	30	300	200	550	700	**0.001**	Real	–
Kmax_3e-3	1.0	(-5, 5)	0	30	300	200	550	700	**0.003**	Real	–
DeepThermo	1.0	(-5, 5)	0	30	300	200	**750**	**1000**	–	Real	–
DeepWind_2	1.0	**(-2, 2)**	0	30	300	200	**750**	**1000**	–	Real	–
DeepWind_8	1.0	**(-8, 8)**	0	30	300	200	**750**	**1000**	–	Real	–
DeepHbed_0	1.0	(-5, 5)	0	30	**0**	200	**750**	**1000**	–	Real	–
Htr_0	1.0	(-5, 5)	0	30	300	**0**	550	700	–	Real	–
Htr_0Hbed_0	1.0	(-5, 5)	0	30	**0**	**0**	550	700	–	Real	–
Htr_0Deep	1.0	(-5, 5)	0	30	300	**0**	**750**	**1000**	–	Real	–
Htr_0Hbed_0Deep	1.0	(-5, 5)	0	30	**0**	**0**	**750**	**1000**	–	Real	–
Melt_0	1.0	(-5, 5)	0	30	300	200	550	700	–	**Pseudo**	**0**
Melt_8	1.0	(-5, 5)	0	30	300	200	550	700	–	**Pseudo**	**8/Nt**
Melt_16	1.0	(-5, 5)	0	30	300	200	550	700	–	**Pseudo**	**16/Nt**
Melt_24	1.0	(-5, 5)	0	30	300	200	550	700	–	**Pseudo**	**24/Nt**

Table S1. List of experiments. List of experiments. The reference simulation and perturbed parameters are marked in boldface. h_{i0} is the initial sea ice thickness across the domain, and the restoring sea ice thickness at the eastern, western, and northern boundaries. U_{a0} and V_{a0} are the zonal and meridional wind extrema at the southern boundary, respectively. The magnitude of the wind speed decreases linearly offshore. A_{tide} is the prescribed tidal current amplitude at the northern boundary. W_{tr} is the half-width of the trough. H_{bed} is the change in bedrock elevation from the shelf break to the southern boundary. H_{tr} is the depth of the trough over the continental shelf. At the zonal boundaries, Z_{sb} is the depth of maximum potential temperature at the shelf break. The depth of maximum potential temperature at the northern and southern boundaries are Z_n (Z_n = 350m in all simulations) and Z_s, respectively. Larger values correspond to a deeper thermocline (Fig. 1D). $\kappa^{3D}{}_{max}$ is the maximum value of the 3D vertical diffusivity. ω_{melt} is the equivalent basal melt rate prescribed at the tilted surface of the pseudo-ice shelf (0, 8, 16, and 24 m/yr, respectively). Nt = 3.0672×10^7 is the number of seconds per year. The names of the experiments in Fig. 3 and fig. S4 are consistent with the symbols in table S1.

REFERENCES

1. F. S. Paolo, H. A. Fricker, L. Padman. Volume loss from Antarctic ice shelves is accelerating. *Science*, **348**, 327–331 (2015).

2. Y. Liu, J. C. Moore, X. Cheng, R. M. Gladstone, J. N. Bassis, H. Liu, J. Wen, F. Hui. Ocean-driven thinning enhances iceberg calving and retreat of Antarctic ice shelves. *Proc. Natl. Acad. Sci. U.S.A.*, **112**, 3263–3268 (2015).

3. E. Rignot, S. Jacobs, J. Mouginot, B. Scheuchl. Ice-shelf melting around Antarctica. *Science*, **341**, 266–270 (2013).

4. H. Pritchard, S. R. Ligtenberg, H. A. Fricker, D. G. Vaughan, M. R. van den Broeke, L. Padman. Antarctic ice-sheet loss driven by basal melting of ice shelves. *Nature*, **484**, 502–505 (2012).

5. S. S. Jacobs, A. Jenkins, C. F. Giulivi, P. Dutrieux. Stronger ocean circulation and increased melting under Pine Island Glacier ice shelf. *Nat. Geosci.* **4**, 519–523 (2011).

6. G. H. Gudmundsson, F. S. Paolo, S. Adusumilli, H. A. Fricker. Instantaneous Antarctic ice sheet mass loss driven by thinning ice shelves. *Geophys. Res. Lett.*, **46**, 13903–13909 (2019).

7. R. M. DeConto, Coauthors. The Paris Climate Agreement and future sea-level rise from Antarctica. *Nature*, **593**, 83–89 (2021).

8. I. Joughin, D. Shapero, P. Dutrieux, B. Smith. Ocean-induced melt volume directly paces ice loss from Pine Island Glacier. *Sci. Adv.* **7**, eabi5738 (2021).

9. I. Joughin, R. B. Alley. Stability of the West Antarctic ice sheet in a warming world. *Nat. Geosci.* **4**, 506–513 (2011).

10. D. P. Walker, A. Jenkins, K. M. Assmann, D. R. Shoosmith, M. A. Brandon. Oceanographic observations at the shelf break of the Amundsen Sea, Antarctica. *J. Geophys. Res.: Oceans*. **118**, 2906–2918 (2013).

11. J. E. Hazel, A. L. Stewart. Are the near-Antarctic easterly winds weakening in response to enhancement of the Southern Annular Mode? *J. Climate*, **32**, 1895–1918 (2019).

12. I. Robinson. Tidal vorticity and residual circulation. *Deep-Sea Res. I: Oceanogr. Res. Pap.* **28**, 195–212 (1981).

13. A. Silvano, P. R. Holland, K. A. Naughten, O. Dragomir, P. Dutrieux, A. Jenkins, Y. Si, A. L. Stewart, B. P. Molino, G. W. Janzing, T. S. Dotto, Alberto C. Naveira Garabato, Baroclinic ocean response to climate forcing regulates decadal variability of ice-shelf melting in the Amundsen Sea. *Geophys. Res. Lett.* **49**, e2022GL100646 (2022).

14. S. Kimura, A. Jenkins, H. Regan, P. R. Holland, K. M. Assmann, D. B. Whitt, M. Van Wessem, W. J. van de Berg, C. H. Reijmer, P. Dutrieux, Oceanographic controls on the variability of ice-shelf basal melting and circulation of glacial meltwater in the Amundsen Sea Embayment, Antarctica. *J. Geophys. Res. Oceans* **122**, 10131–10155 (2017).

15. M. Azaneu, B. Webber, K. J. Heywood, K. M. Assmann, T. S. Dotto, E. P. Abrahamsen. Influence of shelf break processes on the transport of warm waters onto the eastern Amundsen Sea continental shelf. *J. Geophys. Res. Oceans* **128**, e2022JC019535 (2023).

16. K. A. Naughten, P. R. Holland, P. Dutrieux, S. Kimura, D. T. Bett, A. Jenkins. Simulated twentieth-century ocean warming in the Amundsen Sea, West Antarctica. *Geophys. Res. Lett.* **49**, e2021GL094566 (2022).

17. N. C. Jourdain, J.-M. Molines, J. Le Sommer, P. Mathiot, J. Chanut, C. de Lavergne, G. Madec. Simulating or prescribing the influence of tides on the Amundsen Sea ice shelves. *Ocean Model.* **133**, 44–55 (2019).

18. R. Robertson. Tidally induced increases in melting of Amundsen Sea ice shelves. *J. Geophys. Res. Oceans* **118**, 3138–3145 (2013).

19. T. S. Dotto, A. C. N. Garabato, A. K. Wåhlin, S. Bacon, P. R. Holland, S. Kimura, M. Tsamados, L. Herraiz-Borreguero, O. Kalén, A. Jenkins, Control of the oceanic heat content of

the Getz-Dotson Trough, Antarctica, by the Amundsen Sea Low. *J. Geophys. Res. Oceans* **125**, e2020JC016113 (2020).

20. T. S. Dotto, A. C. N. Garabato, S. Bacon, P. R. Holland, S. Kimura, Y. L. Firing, M. Tsamados, A. K. Wåhlin, A. Jenkins, Wind-driven processes controlling oceanic heat delivery to the Amundsen Sea, Antarctica. *J. Phys. Oceanogr.* **49**, 2829–2849 (2019).

21. P. R. Holland, T. J. Bracegirdle, P. Dutrieux, A. Jenkins, E. J. Steig. West Antarctic ice loss influenced by internal climate variability and anthropogenic forcing. *Nat. Geosci.* **12**, 718–724 (2019).

22. M. Donat-Magnin, N. C. Jourdain, P. Spence, J. Le Sommer, H. Gall'ee, G. Durand, Ice-shelf melt response to changing winds and glacier dynamics in the Amundsen Sea sector, Antarctica. *J. Geophys. Res. Oceans* **122**, 10206–10224 (2017).

23. C. M. Little, A. Gnanadesikan, M. Oppenheimer. How ice shelf morphology controls basal melting. *J. Geophys. Res. Oceans* **114**, C12007 (2009).

24. P. Dutrieux, J. De Rydt, A. Jenkins, P. R. Holland, H. K. Ha, S. H. Lee, E. J. Steig, Q. Ding, E. P. Abrahamsen, M. Schröder. Strong sensitivity of Pine Island ice-shelf melting to climatic variability. *Science* **343**,174–178 (2014).

25. P. Mathiot, A. Jenkins, C. Harris, G. Madec. Explicit representation and parametrised impacts of under ice shelf seas in the z^* coordinate ocean model NEMO 3.6. *Geosci. Model Dev.***10**, 2849–2874 (2017).

26. A. T. Bradley, D. T. Bett, P. Dutrieux, J. De Rydt, P. R. Holland. The influence of Pine Island Ice Shelf calving on basal melting. *J. Geophys. Res. Oceans*, **127**, e2022JC018621 (2022).

27. N. C. Jourdain, P. Mathiot, N. Merino, G. Durand, J. Le Sommer, P. Spence, P. Dutrieux, G. Madec. Ocean circulation and sea-ice thinning induced by melting ice shelves in the Amundsen Sea. *J. Geophys. Res. Oceans* **122**, 2550–2573 (2017).

28. R. Chen, J. C. McWilliams, L. Renault. Momentum Governors of California Undercurrent Transport. *J. Phys. Oceanogr.* **51**, 2915–2932 (2021).

29. J. Li, J. Gan. On the formation dynamics of the north equatorial undercurrent. *J. Phys. Oceanogr.* **50**, 1399–1415 (2020).

30. J. Schaffer, R. Timmermann, J. E. Arndt, S. S. Kristensen, C. Mayer, M. Morlighem, D. Steinhage, A global, high-resolution data set of ice sheet topography, cavity geometry, and ocean bathymetry. *Earth Syst. Sci. Data* **8**, 543–557(2016).

31. A. F. Thompson, A. L. Stewart, P. Spence, K. J. Heywood. The Antarctic Slope Current in a changing climate. *Rev. Geophys.* **56**, 741–770 (2018).

32. A. Jenkins, P. Dutrieux, S. Jacobs, E. J. Steig, G. H. Gudmundsson, J. Smith, K. J. Heywood. Decadal ocean forcing and Antarctic ice sheet response: Lessons from the Amundsen Sea. *Oceanography* **29**, 106–117 (2016).

33. P. M. Hampson, J. M. Pringle. Glacial troughs enhance shelf/slope exchange in the barotropic limit. *J. Geophys. Res. Oceans* **127**, e2021JC018207 (2022).

34. S. Adusumilli, H. A. Fricker, B. Medley, L. Padman, M. R. Siegfried. Interannual variations in meltwater input to the Southern Ocean from Antarctic ice shelves. *Nat. Geosci.* **13**, 616–620 (2020).

35. A. F. Thompson, K. G. Speer, L. M. Schulze Chretien. Genesis of the Antarctic slope current in West Antarctica. *Geophys. Res. Lett.* **47**, e2020GL087802 (2020).

36. M. M. Flexas, A. F. Thompson, M. P. Schodlok, H. Zhang, K. Speer. Antarctic Peninsula warming triggers enhanced basal melt rates throughout West Antarctica. *Sci. Adv.* **8**, eabj9134 (2022).

37. A. L. Stewart, J. C. McWilliams, A. Solodoch. On the role of bottom pressure torques in wind-driven gyres. *J. Phys. Oceanogr.* **51**, 1441–1464 (2021).

38. D. T. Bett, P. R. Holland, A. C. Naveira Garabato, A. Jenkins, P. Dutrieux, S. Kimura, A. Fleming. The impact of the Amundsen Sea freshwater balance on ocean melting of the West Antarctic Ice Sheet. *J. Geophys. Res. Oceans*, **125**, e2020JC016305 (2020).

39. A. K. Wåhlin, O. Kalén, K. M. Assmann, E. Darelius, H. K. Ha, T. W. Kim, S. H. Lee. Subinertial oscillations on the Amundsen Sea Shelf, Antarctica, *J. Phys. Oceanogr.* **46**, 2573–2582 (2016).

40. M. Haigh, P. R. Holland, A. Jenkins. The influence of bathymetry over heat transport onto the Amundsen Sea continental shelf. *J. Geophys. Res. Oceans* **128**, e2022JC019460 (2023).

41. P. St-Laurent, J. M. Klinck, M. S. Dinniman. On the role of coastal troughs in the circulation of warm circumpolar deep water on Antarctic shelves. *J. Phys. Oceanogr.* **43**, 51–64 (2013).

42. J. Towns, T. Cockerill, M. Dahan, I. Foster, K. Gaither, A. Grimshaw, V. Hazlewood, S. Lathrop, D. Lifka, G. D. Peterson, R. Roskies, J. R. Scott, N. Wilkins-Diehr, XSEDE: Accelerating Scientific Discovery. *Comput. Sci. Eng.* **16**, 62–74 (2014).

43. J. Marshall, A. Adcroft, C. Hill, L. Perelman, C. Heisey. A finite-volume, incompressible Navier Stokes model for studies of the ocean on parallel computers. *J. Geophys. Res.* **102**, 5753–5766 (1997).

44. J. Marshall, C. Hill, L. Perelman, A. Adcroft. Hydrostatic, quasi-hydrostatic, and nonhydrostatic ocean modeling. *J. Geophys. Res.* **102**, 5733–5752 (1997).

45. T. J. McDougall, D. R. Jackett, D. G. Wright, R. Feistel. Accurate and computationally efficient algorithms for potential temperature and density of seawater. *J. Atmos. Oceanic Tech.* **20**, 730–741 (2003).

46. W. D. Hibler, III. A dynamic thermodynamic sea ice model. *J. Phys. Oceanogr.* **9**, 815–846 (1979).

47. W. D. Hibler, III. Modeling a variable thickness sea ice cover. *Mon. Weather Rev.* **108**, 1943–1973 (1980).

48. M. Winton. A reformulated three-layer sea ice model. *J. Atmos. Oceanic Tech.* **17**, 525–531 (2000).

49. M. Losch, D. Menemenlis, J.-M. Campin, P. Heimbach, C. Hill, On the formulation of sea-ice models. Part 1: Effects of different solver implementations and parameterizations. *Ocean Model.* **33**, 129–144 (2010).

50. D. M. Holland, A. Jenkins. Modeling thermodynamic ice–ocean interactions at the base of an ice shelf. *J. Phys. Oceanogr.* **29**, 1787–1800 (1999).

51. D. E. Gwyther, K. Kusahara, X. S. Asay-Davis, M. S. Dinniman, B. K. Galton-Fenzi. Vertical processes and resolution impact ice shelf basal melting: A multi-model study. *Ocean Model.* **147**, 101569 (2020).

52. R. A. Locarnini, Coauthors. World Ocean Atlas 2023, Volume 1: Temperature (2023).

53. E. Randall-Goodwin, M. P. Meredith, A. Jenkins, P. L. Yager, R. M. Sherrell, E. P. Abrahamsen, R. Guerrero, X. Yuan, R. A. Mortlock, K. Gavahan, A.-C. Alderkamp, H. Ducklow, R. Robertson, S. E. Stammerjohn, Freshwater distributions and water mass structure in the Amundsen Sea Polynya region, Antarctica. *Elem. Sci. Anth.* **3**, 000065 (2015).

54. Y. Si, A. Stewart, I. Eisenman. Coupled ocean/sea ice dynamics of the Antarctic Slope Current driven by topographic eddy suppression and sea ice momentum redistribution. *J. Phys. Oceanogr.* **52**, 1563–1589 (2022).

55. J. Smagorinsky. General circulation experiments with the primitive equations. *Mon. Weather Rev.* **91**, 99–164 (1963).

56. J. Smagorinsky. Some historical remarks on the use of nonlinear viscosities. Evolution of Physical Oceanography, B. Galperin, S. Orszag, Eds., Cambridge University Press, 3–36 (1993).

57. S. M. Griffies, R. W. Hallberg. Biharmonic friction with a Smagorinsky-like viscosity for use in large-scale eddy-permitting ocean models. *Mon. Weather Rev.* **128**, 2935–2946 (2000).

58. R. M. Scott, J. A. Brearley, A. C. Naveira Garabato, H. J. Venables, M. P. Meredith. Rates and mechanisms of turbulent mixing in a coastal embayment of the West Antarctic Peninsula. *J. Geophys. Res. Oceans* **126**, e2020JC016861 (2021).

59. J. A. Brearley, M. P. Meredith, A. C. N. Garabato, H. J. Venables, M. E. Inall: Controls on turbulent mixing on the West Antarctic Peninsula shelf. *Deep-Sea Res. II: Top. Stud. Oceanogr.* **139**, 18–30 (2017).

60. S. Howard, J. Hyatt, L. Padman. Mixing in the pycnocline over the western Antarctic Peninsula shelf during Southern Ocean GLOBEC. *Deep-Sea Res. II: Top. Stud. Oceanogr.* **51**, 1965–1979 (2004).

61. J. W. Loder. Topographic rectification of tidal currents on the sides of Georges Bank. *J. Phys. Oceanogr.*, **10**, 1399–1416 (1980).

62. T. W. K. Armitage, R. Kwok, A. F. Thompson, G. Cunningham. Dynamic topography and sea level anomalies of the Southern Ocean: Variability and teleconnections. *J. Geophys. Res. Oceans*, **123**, 613–630 (2018).

63. S. A. Good, M. J. Martin, N. A. Rayner. En4: Quality controlled ocean temperature and salinity profiles and monthly objective analyses with uncertainty estimates. *J. Geophys. Res. Oceans* **118**, 6704–6716 (2013).

64. A. Jenkins, D. Shoosmith, P. Dutrieux, S. Jacobs, T. W. Kim, S. H. Lee, H. K. Ha, S. Stammerjohn. West Antarctic Ice Sheet retreat in the Amundsen Sea driven by decadal oceanic variability. *Nat. Geosci.* **11**, 733–738 (2018).

65. C. Amante, B. W. Eakins, ETOPO1, 1 Arc-Minute Global Relief Model. Procedures, Data Sources and Analysis. NOAA Technical Memorandum NESDIS NGDC-24. National Geophysical Data Center, NOAA (2009).
